# Utilization of antenatal care among immigrant women in Norway: a nationwide register-based cohort study

**DOI:** 10.1186/s12884-025-07519-x

**Published:** 2025-04-10

**Authors:** Torill A. Rotevatn, Nina Høy-Petersen, Lema Hussaini, Siri E. Håberg, Maria C. Magnus, Nils-Halvdan Morken, Knut-Arne Wensaas, Eva Marie Flaathen, Rannveig Kaldager Hart

**Affiliations:** 1 Division of Health Services, Norwegian Institute of Public Health, PO Box 222, Skøyen, Oslo 0213 Norway; 2 Division of Mental and Physical Health, Norwegian Institute of Public Health, PO Box 222, Skøyen, Oslo 0213 Norway; 3 Centre for Fertility and Health, Norwegian Institute of Public Health, PO Box 222, Skøyen, Oslo 0213 Norway; 4 Department of Global Public Health and Primary Care, University of Bergen, PO Box 7804, Bergen, 5020 Norway; 5 Department of Obstetrics and Gynecology, Haukeland University Hospital, PO Box 1400, Bergen, 5021 Norway; 6 Department of Clinical Science, University of Bergen, PO Box 7804, Bergen, 5020 Norway; 7 Research Unit for General Practice, NORCE Norwegian Research Centre AS, PO Box 7810, Bergen, 5020 Norway; 8 Department of Nursing and Health Promotion, Faculty of Health Sciences, Oslo Metropolitan University, St. Olavs plass, PO Box 4, Oslo, 0130 Norway; 9 Department of Health Management and Health Economics, University of Oslo, PO Box 1089, Blindern, Oslo 0317 Norway

**Keywords:** Antenatal care, Immigrants, Health care utilization, Observational study, Norway

## Abstract

**Background:**

International research suggests that immigrants face poorer access to antenatal care, but comprehensive nationwide studies identifying variations across immigrant groups are lacking. Using national registries like the Medical Birth Registry, we compared antenatal care utilization among immigrant women by country/region of origin to Norwegian women.

**Methods:**

We included 348,547 singleton births between 2012–2018 by women aged ≥ 16 years registered with ≥ 1 antenatal consultation in primary care, including 79,671 (22.9%) births by immigrant women. We calculated odds ratios (OR) and 95% confidence intervals (CI) using both crude and adjusted logistic regression models, assessing the likelihood of immigrant women having fewer consultations than recommended by national guidelines compared to Norwegian women per trimester. Estimates were adjusted for relevant sociodemographic variables.

**Results:**

Large country-specific differences in estimates were noted across all trimesters. In the crude models, Eritrean (OR 3.01 [95%CI: 2.76–3.28]), Somali (OR 2.63 [95%CI: 2.48–2.79]) and Ethiopian (OR 1.90 [95%CI: 1.67–2.16]) women, and women from other Sub-Saharan countries (OR 1.92 [95%CI: 1.77–2.08]), had the highest odds of initiating antenatal care later than the first trimester. In later trimesters, care utilization by immigrants and Norwegian women were more similar, except for lower utilization among Somali women. Sociodemographic variables explained much of the observed differences.

**Conclusion:**

Late initiation and substandard utilization of antenatal care among certain immigrant groups exists in Norway. Timely access to antenatal care is important for maternal and child health. Efforts should be initiated to facilitate earlier initiation of antenatal care, particularly among Eritrean, Somali, Ethiopian and other Sub-Saharan women.

**Supplementary Information:**

The online version contains supplementary material available at 10.1186/s12884-025-07519-x.

## Introduction

In 2023, more than one in four children born in Norway had foreign-born mothers [1, 2]. Despite the large variations between and within origin-countries, studies from several countries have shown that immigrant women are generally at a greater risk of certain complications during pregnancy, childbirth, and the postnatal period [3–5]. Sufficient antenatal care is associated with lower risks of maternal and perinatal complications [6–8], while late initiation of care is associated with preterm birth, low birth weight, fetal and neonatal death, stillbirth and restricted fetal growth [9]. Providing adequate and timely antenatal care for immigrant women is therefore of particular importance to ensure early detection of physical, mental and reproductive health problems throughout pregnancy. However, a comprehensive umbrella review of international research has revealed that immigrants – refugees and asylum seekers in particular – generally experience poorer access to antenatal care [3].

To adapt the antenatal care programme to a more complex and diverse birthing population, there is a need for updated and detailed knowledge of immigrant women’s utilization of these services. The primary care sector is the main provider of antenatal care in Norway. In the current study, we explore differences in the utilization of antenatal care delivered by the primary healthcare sector between immigrant women and Norwegian women using national registries. Past literature reviews of perinatal care utilization and health among immigrants have highlighted the importance of accounting for the effects of various sociodemographic factors to provide better nuance and accuracy to presentations of different groups’ risk profiles [3, 4, 10]. To this end, our study substantially contributes to the existing body of research by exploring differences in antenatal care utilization across maternal countries of origin while including a range of relevant characteristics and using complete population data.

### Literature review

Compared with native-born women, immigrants have poorer access to antenatal care [3], often quantified as having fewer planned antenatal consultations during pregnancy, as well as later care entry [11]. In the Nordic context, where health care is universal and financial barriers to health care are minimal, existing studies suggest a similar pattern. However, these studies are either limited in scope by collapsing multiple origin countries into a broad immigrant category [12], data from a single region only [13] or focus on one immigrant group [14]. In Norway, the only available study concentrates on the period during the COVID- 19 pandemic [15], where substantial mitigation measures were in place, potentially affecting immigrant and native women differently.

In general, the available research indicates a vast degree of origin-specific heterogeneity among immigrant women in terms of their antenatal care utilization. Using data from Malmö in Sweden, Ny et al. [13] reported increased odds of having fewer antenatal visits than recommended among women born in Eastern and Southern Europe, Iraq and Lebanon, and Asia, but not among women born in Western countries and countries in South and Central America, compared with Swedish-born women. Outside the Nordic context, Henderson et al. [16] and Cresswell et al. [17] reported increased odds of late initiation of antenatal care and having fewer antenatal consultations than recommended among immigrants in the UK, although there were substantial differences across ethnicities. The odds of late initiation were particularly increased among African women. In the Netherlands, the odds of inadequate use of antenatal care were increased across the majority of the immigrant sample compared to native Dutch women, with the largest risk increase observed among Moroccan women [18].

The existing literature, both inside and outside the Nordic context, indicates significant disparities in the utilization of antenatal care among women with immigrant background. Responding to the need for updated knowledge that considers the growing diversity of the birthing population, the findings presented by the current research are instrumental for improving women’s access to antenatal care programs and helping reduce social inequalities.

## Materials and methods

### Study design and setting

We conducted a nationwide register-based cohort study using data from national health and administrative databases to compare the utilization of antenatal consultations in primary care among immigrant women, with that among Norwegian women. In Norway, all pregnant women residing in the country, independent of immigrant status, have similar rights to antenatal care and can choose between receiving free-of-charge antenatal care from a general practitioner (GP) and/or a midwife as part of primary municipal healthcare services [19]. The nationally recommended basic program for women with healthy pregnancies during the study period consisted of eight planned consultations, including one routine ultrasound scan in the second trimester, where the first consultation should be in the first trimester between pregnancy weeks 8 and 12 [20]. Table 1 presents an overview of the recommended consultations by trimester and week of gestation.

**Table 1:** Timing of the recommended antenatal consultations and ultrasound scan per trimester

1. trimester (≤ 13w6 d GA)	2. trimester (14w + 0 d – 26w + 6 d GA)	3. trimester (> 27w + 0 d GA)
Consultation 1: GA week 8–12	Ultrasound scan: GA week 17–19 Consultation 2: GA week 24	Consultation 3: GA week 28 Consultation 4: GA week 32 Consultation 5: GA week 36 Consultation 6: GA week 38 Consultation 7: GA week 40

*GA* Gestational age

While consultations are normally offered by the primary care sector, ultrasound scans are typically performed by specialist healthcare services. Additional consultations are offered if needed. Women with high-risk pregnancies often receive specialized antenatal care in addition to services provided by the primary healthcare system, either for specific periods or throughout their pregnancy. After completing 40 weeks of gestation, women receive additional consultations for evaluation of induction.

### Data sources

The population of women was identified via the Population Registry, from which we obtained information on the child month and year of birth, maternal- and partner’s country of birth and birth year, and time of immigration. Information on women’s contacts with GPs and midwives during pregnancy was obtained from the Norwegian Control and Payment of Health Reimbursements Database (KUHR). From KUHR, we acquired tariff codes, diagnosis codes based on the International Classification of Primary Care 2nd Edition (ICPC- 2) and the date of each claim. From the Medical Birth Registry, we obtained information on gestational age at delivery, parity, civil status, multiple pregnancies, ultrasound-estimated delivery date, birthweight by gestational age z-scores, registration of preeclampsia and diabetes status. Gestational age at delivery was based on ultrasound scans performed in the second trimester. From the National Education Database, we collected information on women’s highest achieved education in the year before giving birth. From Statistics Norway, we obtained household income in the year before giving birth and classification of immigration category. Registers were linked using a unique personal identifier given to all Norwegian residents at birth or upon immigration.

### Study population

We identified all live births in Norway between 1 January 2012 and 31 December 2018 to women born between 1970 and 1999, who were 16 years or older at the time of giving birth. The latter as persons over 16 years are of legal age in relation to health service rights in Norway. Both mothers and children had to be registered with a personal identification number, thus including all mothers residing in Norway irrespective of their immigrant background. We included only births by women registered as residents no later than the first day of pregnancy, with at least one antenatal consultation, and with information on gestational age at birth and the ultrasound-estimated delivery date. As our aim was to study the utilization of antenatal consultations for uncomplicated pregnancies in primary care, we excluded pregnancies with severe preeclampsia, pregestational diabetes type 1, and multiple pregnancies (twins/triplets), as these pregnancies are handled to a much greater extent by specialist health care services. For the same reason, we excluded preterm (< 34 gestational weeks) and very postterm births (≥ 43 gestational weeks) and births with small for gestational age babies (birthweight by gestational age z-score ≤ 3). In addition, women who gave birth before gestational week 34 had significantly different preconditions for utilizing antenatal care, as the majority of consultations are planned from gestational week 32 onward. The final sample used in the analysis consisted of 348,547 singleton live births by 263,742 women (see flow chart, figure A1 in Additional file 1), with 79,671 (22.9%) of these births being among immigrant women (*n* = 63,134).

### Pregnancy consultations

To identify pregnancy consultations performed by GPs, we included reimbursement claims registered with tariff codes 217a (*first complete examination and guidance of pregnant women*) and 271b (*subsequent pregnancy control*), as these codes are used for consultations that are part of the national antenatal care program. We additionally included claims coded with tariff code 217c (*pregnancy control performed by a midwife in collaboration with a GP, by a midwife employed at a GP office).* To identify pregnancy consultations performed by midwives, we included claims with the specific midwife tariff code 1a (*first complete examination and guidance of pregnant women*) and tariff code 1b (*pregnancy consultation*). In addition to these pregnancy-specific claims, we included claims from GPs or midwifes registered with the ICPC- 2 code W781 (*pregnancy consultation*).

### Exposure

The main exposure was having immigrant status, defined as women born outside Norway by two foreign-born parents. These women were compared to the rest of the population (hereafter referred to as Norwegian women), which included Norwegian-born women and foreign-born women with at least one Norwegian-born parent. The country of birth denotes the country of origin for immigrants.

### Outcomes

Our main outcome was receiving fewer antenatal consultations than recommended per trimester, as presented in Table 1. For instance, women having no consultations during the first trimester (0w + 1 d GA to 13w + 6 d GA) were identified with the outcome, as at least one consultation is recommended during this period. By grouping consultations by trimesters, the outcomes were not affected by situations where underutilization in one part of the pregnancy was potentially outweighed by extra consultations in another part of the pregnancy. Additionally, this broad grouping of consultations prevented noise, such as holidays, from affecting the outcomes.

We summarized the number of consultations per trimester for each pregnancy and created a dummy variable indicating whether women had fewer consultations than the recommended number of consultations per trimester. We counted the number of antenatal consultations starting from the first day of pregnancy up to either i) the date of birth or ii) up to seven days after the estimated due date for women who were still pregnant at that time. The latter approach was used to avoid including additional consultations related to postterm deliveries. We estimated the delivery date on the basis of gestational age at birth and ultrasound-estimated due date, with a term pregnancy being 283 days according to the Norwegian Directorate of Health [21]. We identified the first day of pregnancy by subtracting the gestational age at birth from the delivery date. In a limited number of cases, women had consultations with a GP and a midwife on the same day. Only one consultation per day was included when the outcomes were constructed. To account for shorter pregnancies with less time to attend all check-ups, gestational age at birth was considered when the outcome in the third trimester was defined. If women gave birth 1) before 37w + 0 d GA (gestational age), 2) between 37w + 0 d and 38w + 6 d GA, 3) between 39w + 0 d and 40w + 6 d GA or 4) after 40w + 6 d GA, she is recommended to have attended at least two, three, four and five consultations during this trimester, respectively.

### Covariates

We included a range of sociodemographic factors related to the potential barriers to seeking and utilizing antenatal care among immigrant women [3, 11, 22, 23]. We calculated the period of residency before pregnancy from the date of immigration to the first day of pregnancy for immigrant women. We grouped periods of residency as: *0–2*, *3–5*, *6–9*, *10–15* and > *15* years. Immigrant women with at least 15 years of residency were grouped with Norwegian women. Age was categorized into five-year bins based on the hypothesis of a non-linear relationship to the outcomes. Civil status was categorized as *married/registered partner*, *cohabiting, no partner* (unmarried, single, divorced, separated, widow) and *other/unknown*. Maternal education attainment was categorized as *primary school*, *upper secondary school*, *bachelor’s degree or equivalent*, *masters’ degree or equivalent,* or *missing*. Missing data were treated as a separate category, as a significant proportion of immigrants had their education abroad and were not included in the Norwegian databases. Household income, adjusted for the number of children in the household, was categorized into quartiles (q1: ≤ *266,362 NOK, q2: 266,363–362,602 NOK, q3: 362,603–464,065 NOK, q4:* ≥ *464,066 NOK*). Missing (*n* = 5,756) and negative (*n* = 279) income was recoded as 0. Missing income was correlated with a short residency period and was retained in the analyses to avoid excluding many immigrants. Parity denoted the number of previously born children for each pregnancy: *0 children*, *1 child*, *2 children*, *3 children* and ≥ *4 children*. Finally, the partner’s origin was categorized as *Norwegian-born*, *foreign-born* or *father unknown*.

### Statistical analyses

We used logistic regression models to estimate odds ratios (OR) with 95% confidence intervals (CI). ORs represents the odds of an outcome event in one group divided by the odds of the event in another group [24]. In this study, ORs denote the odds of having fewer than the recommended number of consultations per trimester when comparing immigrant women and their country of origin to Norwegian women. Norwegian women were set as the reference category, under the assumption that women with a Norwegian background possess the requisite understanding of the Norwegian healthcare system and the language and navigation skills needed to access antenatal care. Thus, the comparison was the odds of having fewer consultations than recommended for immigrant women compared with Norwegian women. We calculated estimates per origin country with ≥ 1000 births in the sample and per Global Burden of Disease region for countries with < 1000 births in the sample [25]. The regions are categorized based on epidemiological similarity and geographical closeness. European subregions (central, eastern and western) were kept retaining detailed analyses. The Central Asia subregion was grouped together with Southeast Asia, East Asia and Oceania, as relatively few women originated from these regions.

We estimated two main models: 1) unadjusted models and 2) models adjusted for sociodemographic factors: year of delivery, maternal age, parity, civil status, maternal educational attainment, household income, partner origin, and period of residency. Estimates in the adjusted models demonstrate how variance in sociodemographic factors influences outcomes, which helps elucidate potential mechanisms in the relationship between country of origin and the utilization of antenatal care. All women were included in these models, with a variable indicating country/region of origin. For a more detailed interpretation of the potential mechanisms related to the covariates, we additionally conducted analyses using three different adjusted models: 1) models adjusted for demographic factors (year of delivery, maternal age, parity and civil status), 2) models adjusted for socioeconomic factors (maternal educational attainment and household income), and 3) models adjusted for factors related to immigration (partner origin and period of residency). We additionally ran subgroup analyses including only nulliparous women, as it is of particular importance that these women receive an adequate level of antenatal care. Standard errors were clustered at the maternal level to account for the potential correlation of observations within mothers registered with several births in the sample. All the analyses were performed in R version 4.1.2.

### Sensitivity analysis

In the main analysis, we included only health care contacts registered with a code denoting routine pregnancy consultation to be the purpose of the visit. To test the robustness of the main analysis, we reran the main analyses using additional data including claims registered with other ICPC- 2 codes representing conditions or diseases related to pregnancy (Table 1 in Additional file 1), to evaluate the frequency of potentially missed encoding of pregnancy controls when women had pregnancy-related contact with their GP.

## Results

Table 2 presents the background characteristics of the cohort consisting of 348,547 singleton live births, 79,671 (22.9%) by immigrant women and 268,876 by Norwegian women. Compared to Norwegian women, pregnant of immigrant background women tended to have higher parity, lower educational attainment and lower household income. The majority of immigrants (54.9%) had resided in Norway for five years or less at the time of delivery. In total, 1,515,934 antenatal consultations were conducted by GPs and 1,464,413 by midwives. A greater proportion of consultations were conducted by general practitioners in the immigrant population (55.0%) than in the population of Norwegian women (49.3%). A total of 2,951,218 consultations were included in the analysis. The median number of consultations per pregnancy was 9 for both immigrants and Norwegian women, with slightly wider interquartile range for immigrants (interquartile range: 7–12) than for Norwegians (interquartile range: 7–11).

**Table 2:** Maternal characteristics, consultations and outcomes per pregnancy, by immigrant status, Norway 2012–2018

	**Immigrant women**	**Norwegian women**	
	(*n* = 79 671)	(*n* = 268 876)	*p*-value
**Maternal characteristics**			
Age			
16–20 years	1 065 (1.3)	6 658 (2.5)	
21–25 years	9 935 (12.5)	44 844 (16.7)	
26–30 years	27 394 (34.4)	96 645 (35.9)	
31–35 years	27 378 (34.4)	83 234 (31.0)	
≥ 36 years	13 899 (17.4)	37 495 (13.9)	< 0.001
Parity			
0 children	30 292 (38.0)	113 764 (42.3)	
1 child	29 798 (37.4)	102 807 (38.2)	
2 children	12 293 (15.4)	40 262 (15.0)	
3 children	4 418 (5.5)	8 842 (3.3)	
≥ 4 children	2 870 (3.6)	3 201 (1.2)	< 0.001
Educational attainment			
Primary school	17 042 (21.4)	44 237 (16.5)	
Upper secondary school	12 403 (15.6)	69 320 (25.8)	
Bachelor’s degree or equivalent	16 541 (20.8)	111 806 (41.6)	
Masters’ degree or equivalent	12 171 (15.3)	42 805 (15.9)	
Missing	21 514 (27.0)	708 (0.3)	< 0.001
Household income			
Lowest quartile	38 246 (48.0)	49 313 (18.3)	
Mid-lowest quartile	18 749 (23.5)	67 417 (25.1)	
Mid-highest quartile	12 116 (15.2)	74 337 (27.6)	
Highest quartile	10 560 (13.3)	77 809 (28.9)	< 0.001
Civil status			
Married/registered partner	57 863 (72.6)	93 423 (34.7)	
Cohabiting	15 861 (19.9)	160 114 (59.5)	
No partner	4 729 (5.9)	12 859 (4.8)	
Other/unknown	1 218 (1.5)	2 480 (0.9)	< 0.001
Period of residency			
≤ 2 years	22 052 (27.7)	0 (0)	
> 2—≤ 5 years	21 708 (27.2)	0 (0)	
> 5—≤ 10 years	19 213 (24.1)	0 (0)	
10—≤ 15 years	7 882 (9.9)	0 (0)	
> 15 years or Norwegian women	8 816 (11.1)	268 876 (100)	< 0.001
Partners origin			
Norwegian-born	21 367 (26.8)	239 321 (89.0)	
Foreign-born	53 884 (67.6)	24 002 (8.9)	
Unknown	4 420 (5.5)	5 553 (2.1)	< 0.001
**Consultation characteristics**			
Number of consultations			
GP	376 507 (55.0)	1 125 087 (49.3)	
Midwife	304 624 (44.5)	1 145 449 (50.2)	
GP and midwife on the same day	3 966 (0.6)	10 374 (0.5)	
In total	685 097 (100)	2 280 910 (100)	< 0.001
Number of consultations per pregnancy, *median (q1 – q3)*			
GP	5 (3–8)	4 (2–7)	< 0.001
Midwife	4 (2–6)	5 (3–7)	< 0.001
Total contacts^a^	9 (7–12)	9 (7–11)	< 0.001
**Outcomes**			
Fewer consultations than recommended in 1. trimester	17 635 (22.1)	45 622 (17.0)	< 0.001
Fewer consultations than recommended in 2. trimester	5 411 (6.8)	17 715 (6.6)	0.04
Fewer consultations than recommended in 3. trimester	19 017 (23.9)	60 605 (22.5)	< 0.001

Number of observations (column percentage) are presented unless otherwise specified

*Abbreviations*: *GP* General practitioner, *q1 – q3* interquartile range

^a^Including only one consultation per day

*P*-values derived using chi-square test for categorical variables and Wilcoxon rank sum test for medians

### Late initiation of antenatal care

The median gestational day for the first registered consultation was day 78 for immigrants and day 76 for Norwegian women. The median gestational day for the first registered consultation was day 90 for Eritrean women and day 85 for Somali women. Not having any consultations during the first trimester denotes initiation of antenatal care later than recommended (late initiation), which occurred in a larger proportion of pregnancies by the immigrant population (22.1%) than by the population of Norwegian women (17.0%) (Table 2). Late initiation was observed in 38% of the pregnancies of Eritrean women and in 35% of Somali women (table 2 in Additional file 1). In addition, very late initiation of antenatal care (after week 19 of pregnancy) was registered in 14% of the pregnancies by Eritrean women and in 12% of the pregnancies by Somali women, compared with 6% in the population of Norwegian women.

**Fig. 1:**
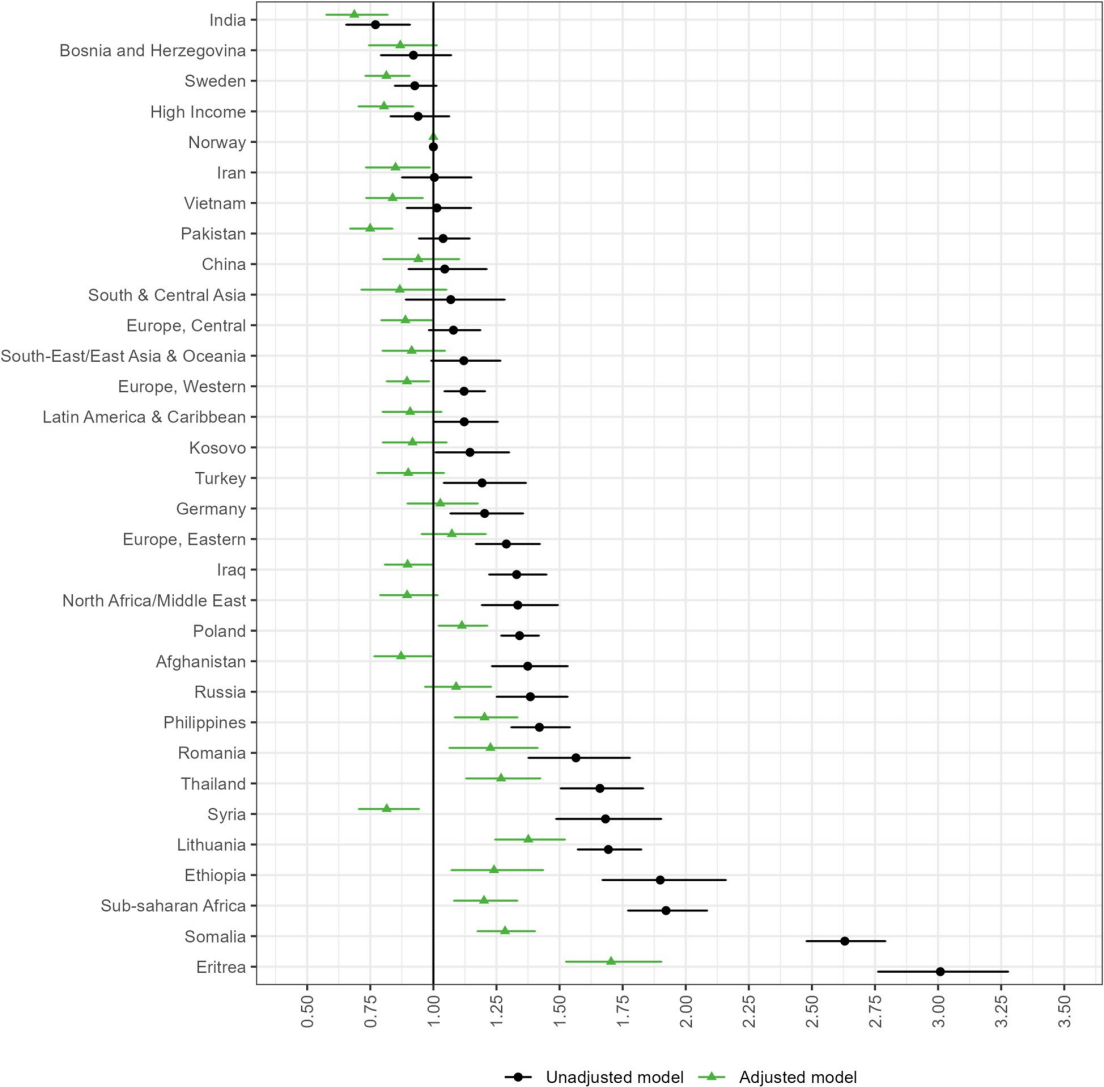
Odds ratios (95% confidence intervals) of not having any antenatal consultations during the first trimester. Estimates are presented by country/region of origin, compared with Norwegian women. The adjusted model includes child birth year, parity, age, civil status, maternal educational attainment, household income, partner’s origin and period of residency. The label “High income” comprises high-income countries located outside Europe

The unadjusted odds ratios of late initiation of antenatal care were considerably higher for most immigrant groups than for Norwegian women (Fig. 1, table A3 in Additional file 1). Notably, elevated ORs were observed for Eritrean (OR 3.01 [95% CI: 2.76–3.28]), Somali (OR 2.63 [95% CI: 2.48–2.79]), and Ethiopian women (OR 1.90 [95% CI: 1.67–2.16]), and women originating from other sub-Saharan African countries (OR 1.92 [95% CI: 1.77–2.08]). Moreover, elevated ORs were also observed among women from specific Eastern-European, Middle Eastern, and Southeast-Asian countries. Adjustment for covariates particularly attenuated the ORs among African and Middle Eastern women, where inclusion of socioeconomic and immigrant-related factors in the model led to substantial attenuation of the difference (figure A2 in Additional file 1). Adjustment for demographic variables, including parity, also led to large reductions in the ORs observed among Somalian and Syrian women. When adjusting for all covariates, the highest odds ratio of late initiation of antenatal care was still observed among Eritrean women (OR 1.70 [95% CI: 1.53–1.90]).

In the fully adjusted model, several covariates showed significant independent associations with late initiation of antenatal care (table A3). The ORs were slightly higher in the last four years of the study period (2015–2018) than at the beginning of the study period (2012). Additionally, the odds of late initiation of antenatal care increased with increasing parity. Compared with women aged 31–35 years, women aged 16–20 years had the highest odds of late initiation. Compared with married women, women without a partner exhibited a notably greater OR of late initiation. Women with the lowest educational attainment and household income had the highest odds of late initiation. Finally, women with a period of residency of two years or less were associated with the highest odds of late initiation compared with the joint group of Norwegian women and immigrants with at least 15 years of residency, and the odds decreased with increasing period of residency.

### Utilization of antenatal care in the second and third trimester

**Fig. 2:**
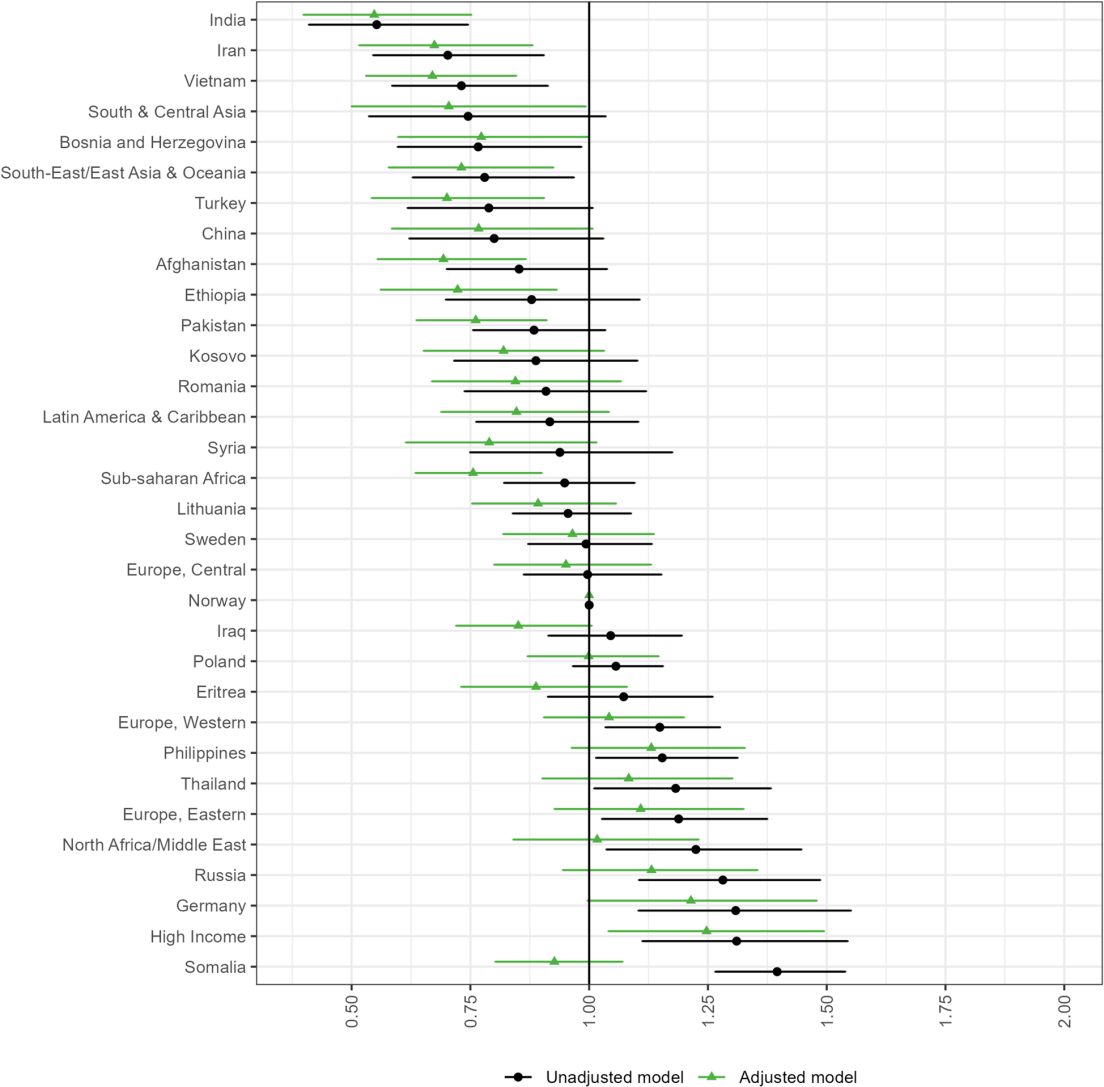
Odds ratios (95% confidence intervals) of having fewer consultations than recommended during the second trimester. Estimates are presented by country/region of origin, compared with Norwegian women. The adjusted model includes child birth year, parity, age, civil status, maternal educational attainment, household income, partner’s origin and period of residency. The label “High income” comprises high-income countries located outside Europe

**Fig. 3:**
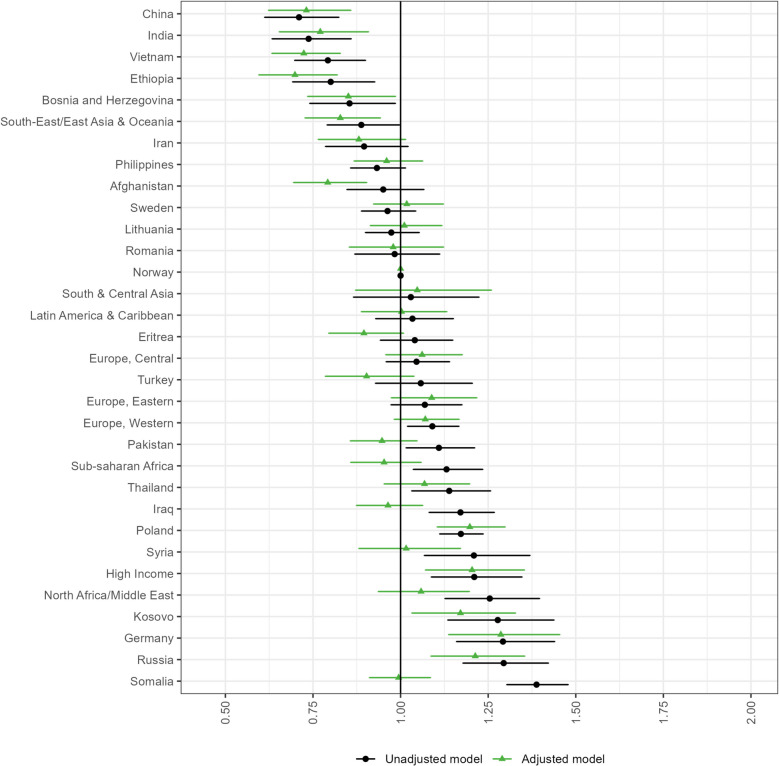
Odds ratios (95% confidence intervals) of having fewer consultations than recommended during the third trimester. Estimates are presented by country/region of origin, compared with Norwegian women. The adjusted model includes child birth year, parity, age, civil status, maternal educational attainment, household income, partner’s origin and period of residency. The label “High income” comprises high-income countries located outside Europe

Compared with Norwegian women, slightly greater proportions of immigrants had fewer consultations than recommended in the second and third trimester (Table 2). The ORs of having fewer consultations than recommended were generally lower in these trimesters than in the first trimester (Figs. 2, 3, table A4-A5 in Additional file 1). The profiles of immigrant women with increased unadjusted odds of having fewer consultations than recommended were also slightly different in these subsequent trimesters. The highest unadjusted odds were observed for Somali women in both the second and third trimester (second trimester: OR 1.40 [95% CI 1.27–1.54], third trimester: OR 1.39 [95% CI 1.30–1.48]), followed by women from high income countries (OR 1.31 [95% CI 1.11–1.54]), German women (OR 1.31 [95% CI 1.10–1.55]) in the second trimester, and Russian (OR 1.29 [95% CI 1.18–1.42]) and German women (OR 1.29 [95% CI 1.16–1.44]) in the third trimester. Covariate adjustments affected the ORs to a smaller extent than it did in the first trimester, but fully attenuated the increased odds observed among Somali women. For these women, immigrant and socioeconomic factors were particularly influential in the second trimester, whereas the introduction of background factors caused the greatest attenuation in the third trimester (figure A3-A4 in Additional file 1). After adjusting for covariates, women from high income countries had the highest ORs of having fewer consultations than recommended in the second trimester (OR 1.25 [95% CI 1.04–1.49]). German women had the highest adjusted OR in the third trimester (OR 1.29 [95% CI 1.14–1.45]). Our data further revealed a weak association between having a period of residency of ≤ 2 years and having fewer consultations than recommended in the second trimester, whereas there were no clear differences between periods of residency and having fewer consultations than recommended in the third trimester.

### Subgroup analyses

When only nulliparous women were included, the results generally mirrored the main results (figures A5-A7 in Additional file 1). However, there were a few exceptions. The odds of not following recommendations were, in some cases, lower for first time mothers than what was observed in the analyses of the main sample. In particular, this was the case for Somali women in all trimesters, for Syrian women in the first trimester, for women from the North Africa/Middle East region in the second trimester, and for Kosovan, Syrian, Iraqi women and North African/Middle Eastern women in the third trimester.

### Sensitivity analyses

When also including claims registered with additional pregnancy-related ICPC- 2 codes, the analyses generally yielded slightly lower ORs for some immigrant groups in the first and third trimester and slightly higher ORs for some groups in the second trimester (figure A8-A10 in Additional file 1). The estimates were, however, largely comparable with the main results.

## Discussion

This study assessed how utilization of the antenatal care program in Norway varies with the mother’s country of origin, using population-based register data to provide new, uniquely nuanced and nationally representative knowledge.

First, and in correspondence with past research from Europe [12–14, 16–18, 26], we found that a larger proportion of immigrant women in Norway initiated antenatal care later than recommended (22.1%) compared with Norwegian women (17.0%). The differences between these overarching groups on median day of gestation for the first consultation were however small and insignificant, while there existed large internal variation among immigrant women across country of origin. The crude ORs of not having any consultation during the first trimester were highest among Eritrean, Ethiopian and Somali women, and among women from other countries in the sub-Saharan region, and these differences were largely explained by sociodemographic factors. In addition, the odds of late initiation of antenatal care were increased for immigrants originating from certain Eastern-European, Middle Eastern and Southeast Asian countries, also after taking sociodemographic factors into account.

Second, although both crude and adjusted odds ratios of having fewer antenatal consultations than recommended were elevated for certain immigrant groups in the second and third trimester, these estimates were markedly lower than those reported in the first trimester. This was particularly the case for women from Eritrea, Ethiopia, and the Sub-Saharan region. The results may thus suggest that once immigrant women have initial pregnancy-related contact with the healthcare system, they tend to follow the antenatal care program similarly to Norwegian women of comparable sociodemographic characteristics. Notably, this trend was not observed among Somali women, as their odds ratios for having fewer consultations than recommended remained consistently high throughout all trimesters.

The strong associations between country of origin and late initiation of antenatal care observed among the majority of immigrants clearly identify late initiation as the key challenge to address in future initiatives aimed at mitigating disparities in antenatal care between immigrants and the population of Norwegian women. In a study examining recently migrated women from low- and middle-income countries residing in Oslo, Bains et al. [27] identified difficulties in navigating the Norwegian maternity healthcare system as one of the primary barriers to accessing optimal maternity care. Approximately half of the study participants reported issues in this area. The results from the current study suggest that navigational challenges may be a significant hindrance to initiating antenatal care in a timely manner. This is notably evidenced by the independent association between shorter durations of residency and higher odds of delayed initiation. Additionally, adjusting for length of residency and partner's origin significantly reduced the odds of late initiation. However, making these adjustments had a smaller impact on the ORs of having fewer consultations than recommended in the later trimesters. Moreover, there was no association between residency duration and outcomes in later trimesters, indicating that once initial engagement in antenatal care is achieved, women with shorter residency periods receive care comparable to that of Norwegian women.

Our study revealed that women who immigrate from typical refugee-sending countries (e.g., Somalia, Eritrea, Ethiopia, and other sub-Saharan nations) had particularly high odds of late initiation of antenatal care compared with Norwegian women, which is in line with findings reported by Leppälä et al. [12] on the utilization of antenatal care among immigrants from conflict-affected areas. These findings could reflect the compounded vulnerabilities faced by women with refugee backgrounds. In addition to system navigation challenges, other structural, organizational, social, personal and cultural barriers can also affect immigrant women’s access to and utilization of antenatal care [3]. For instance, the prevalence of female genital mutilation is high in these countries, which can prevent women from accessing health care services due to stigmatization and low expectations of care [28]. Also, research conducted in Nordic countries links immigrant women’s suboptimal use of antenatal care to challenges with health literacy [29, 30], insufficient provision of health information [30], limited financial and social resources [27, 31] and language barriers [27, 29–32]. Moreover, social isolation, misassumptions that free antenatal care is associated with costs, and mistrust of health professionals may represent additional barriers experienced by refugee women in particular [3]. The significance of socioeconomic barriers could be even more pronounced in refugee families, as they more often lack stability and connection to the labor market and other parts of society [3]. In our research, socioeconomic factors emerged as important explanatory factors for timely initiation among women from typical refugee-sending countries, while these factors had less impact on the adjusted estimates of care utilization in the later trimesters. This could suggest that the antenatal care system succeeds in mitigating the socioeconomic vulnerabilities experienced by these women once they enter care.

Adjusting for a range of sociodemographic factors largely, but not fully, attenuated, the odds of late initiation of antenatal care among women from typical refugee-sending countries. Thus, unexplained variance still exists, which could reflect the impact of other unmeasured mechanisms. To mention three such mechanisms, research suggests that variations in cultural beliefs about pregnancy could contribute to lower utilization of care among these women. Indeed, a recent literature review on African immigrant women’s maternal health care utilization [33] and a study focusing primarily on sub-Saharan refugees in Melbourne [34] both indicate that these populations often view pregnancy and childbirth as safe and normal processes that do not require specialized medical attention. Second, rates of unintended pregnancies are particularly high among Sub-Saharan women [35], which may also be a reason for late care-seeking [36, 37]. Third, country-specific differences in healthcare expectations and experiences of stigma, prejudice, and discrimination, which have also been reported in maternity care in the Nordic context [30], could influence health-seeking behaviors [38]. The effects of these barriers to care remain unquantified in the current study.

As mentioned above, the composition of immigrant groups that stand out with higher odds of completing fewer antenatal consultations than recommended changed from the first to the following trimesters. In the subsequent trimesters, women from select European countries appeared less likely to follow the antenatal care program than Norwegian women did. This may be partly explained by the challenges proposed above, particularly for labor immigrants from Eastern European EU member states, who are not required to complete the Norwegian introduction program where language training and information concerning their rights and available services in Norway is provided [39]. Moreover, women migrating from countries geographically close to Norway may also have greater opportunities to access antenatal care transnationally. Such transnational usage of health care can be related to dissatisfaction with the health care system in their country of residence, unfamiliarity with the health care system, cultural or language barriers, having close family ties in their country of origin, and the ease of cross-border movement [40–42]. For example, studies have shown that East European migrants living in other European host countries continue to use health care services in their home countries to a large extent [43, 44]. Findings from a qualitative study of immigrant women’s experiences with maternal health services in Norway have shown that expectations of care are largely influenced by their knowledge of the systems and practices in their country of origin and that dissatisfaction with *undermedicalized* care involving lower levels of surveillance and specialized care exists [42]. This led some of the women in the mentioned study to access services abroad or in the private sector if they had the means. Both transnational and private use of antenatal care was not possible to include in the current study and would thus manifest as apparent underutilization in our data. For this reason, we expect that some of the underutilization of antenatal care observed among German, Russian and Central/Eastern-European immigrants is explained by transnational health seeking behavior or the utilization of private care.

### Implications for practice

Pregnancy is a time when many foreign-born women first encounter primary health care services, where they may be particularly motivated to make health-related behavioral changes [45]. A positive experience of antenatal care could also motivate new mothers to continue to seek health care for their children and themselves. Securing good experiences with antenatal health care services may therefore have positive effects on the next generation and, more generally, on public health. Receiving early and timely antenatal care reduces maternal and perinatal morbidity and mortality both directly through early detection and treatment of pregnancy-related complications, and indirectly, through the identification of women at increased risk of developing complications during labour and delivery, thus ensuring referral to an appropriate level of care [46, 47]. As such, adjusting antenatal care to meet the needs of a more diverse population is considered an important public health priority.

We observed smaller differences in the utilization of antenatal care between immigrant women and Norwegian women in the second and third trimester than in the first trimester, which could indicate that immigrant women follow the national recommendations for antenatal care once they enter the program. These observations may signify that the Norwegian universal health care system succeeds in providing equitable antenatal care for all women, regardless of their background, at least from when they enter care. Our results highlight the potential for improving the timely initiation of antenatal care, particularly for women of Somali, Eritrean, Ethiopian, and other sub-Saharan origin. However, the large range of potential mechanisms at play makes this a complex challenge. The presence of accessible, comprehensive, appropriate, and meaningful information on patient rights, as well as the organization of the antenatal care program and its benefits, is crucial for empowering immigrant women in making informed choices of antenatal care utilization. Enhancing the multisectoral provision of such information both prior to and during pregnancy may be a starting point to promote timely and continuous antenatal care among immigrants at risk of experiencing barriers in accessing antenatal care. Qualitative studies that explore potential context-specific reasons for late initiation among Somali, Eritrean, Ethiopian, and other sub-Saharan women in Norway are needed to further support the development of effective interventions toward this end.

Unlike other groups in our study, we observed lower utilization of antenatal care than recommended among Somali women in all trimesters. Improving antenatal care utilization continuously throughout pregnancy may therefore be a priority once these women have entered care. Findings from a qualitative study on Somali women’s experiences with antenatal care in Norway pinpoint that trust, continuity and cultural sensitivity in care are important for these women’s willingness to make use of care [48]. Receiving care from a familiar caregiver with appropriate cultural competence may thus be a particularly effective means by which to increase antenatal care utilization among these women. In a qualitative study on the timing of antenatal care initiation in England, Somali interviewees noted that early antenatal care was devaluated by those who had experienced normal and uncomplicated pregnancies [49]. The women interviewed also reported a desire to maintain control over their pregnancy without interference from healthcare professionals, which might reflect their view of pregnancy and childbirth as something natural and normal that does not need medical intervention. These findings underscore the importance of having health care personnel with sufficient cultural competence who are able to build trusting relationships to improve antenatal care utilization. In our study, compared with the full sample, nulliparous Somali women had lower odds of both late initiation of antenatal care and having fewer consultations than recommended in the second and third trimester, showing that the first pregnancy can be a window of opportunity in terms of promoting sufficient utilization of antenatal care in later pregnancies. It is possible that some of these women received additional specialized antenatal care, but this was outside the scope of our study.

### Strengths and limitations

This research is based on high-quality and nationwide registries, which cover a considerable time-period. The study design enables a detailed description of how antenatal consultations delivered by the primary care sector are utilized across immigrant groups, with attention to country-specific variations. Our methodology secures near perfect recruitment of all immigrant groups and sampling representativity, which would be impossible to match using other study designs, such as surveys. The large study population enabled analyses with a high level of granularity and power, thereby allowing the differentiation of various patterns of antenatal care utilization among immigrant women originating from a broad range of countries.

There are, however, some limitations. First, our dataset is not entirely up to date, encompassing pregnancies up to and including 2018. Since that time, the influx of immigrants to Norway has continued [50]. Consequently, future research utilizing updated data would be valuable for assessing how antenatal care services address the challenge of adapting to a more diverse population. Second, we do not have data on the utilization of transnational care-seeking or private care services. Consequently, we were unable to disentangle whether the inadequate utilization of care was indicative of a real deficiency in necessary care provision or if women were receiving these services outside of the primary healthcare sector. Given the availability of free-of-charge services in Norway, the usage of private care services is presumptively limited. When used, private antenatal care services typically serve as an addition to public services, likely by women with different expectations from the national program and the financial means to afford them. Third, administrative registry data do not include data on all relevant factors affecting health care use, e.g. health literacy or language proficiency. As such, the variance remaining in the full model for some origin countries in particular are subject to interpretation. Fourth, immigrants without residential status are not included in the data. Newly arrived immigrants and nonresidents have not yet been provided with the standard Norwegian personal identification number, which is necessary to track individuals’ health care utilization and link data from different national databases with a high degree of accuracy. Furthermore, a significant portion of immigrants lacked data on educational attainment. However, previous application of imputation methods has demonstrated minimal changes in the distribution of educational attainment categories across immigrants [51]. To prevent exclusion of this large proportion of immigrants, we maintained *missing* as a standalone category. Finally, the data quality of KUHR is highly dependent on health personnel correctly registering their activities. However, there exists strong organizational and financial incentives, such as activity-based financial agreements, that most likely enhance the accuracy of activity registration.

## Conclusion

Overall, it seems like the health care system succeeds in providing equitable antenatal care for all women, as the large majority of immigrant women entered and utilized care at levels comparable to Norwegian women. However, late initiation of antenatal care emerges as the main disparity in antenatal care utilization between immigrant and Norwegian women, with women from Ethiopia, Somalia, Eritrea, and other sub-Saharan African countries being particularly vulnerable. In addition, Somali women tend to have fewer consultations than recommended throughout all stages of pregnancy. Delayed entry into, and inadequate utilization of, the antenatal care program may hinder healthcare providers'ability to offer personalized care that meets the specific needs of pregnant women, potentially jeopardizing maternal and child health. Therefore, public health initiatives should focus on reducing potential barriers to entering and utilizing care for at-risk immigrant groups and adjusting care to the needs of a more diverse birthing population.

## Supplementary Information

Additional file 1.

## Data Availability

The datasets generated and analyzed for the current study are not publicly available due to data protection reasons. Researchers wishing to use this data must submit a formal application, which is accessible at https://helsedata.no. This application should outline a comprehensive research proposal, detailing the project’s objectives and methodologies. Approval from the Regional Committees for Medical and Health Research Ethics is required, application for approval is available at rekportalen.no.

## Abbreviations

OR: Odds ratio
CI: Confidence interval
GP: General practitioner
GA: Gestational age
KUHR: The Norwegian Control and Payment of Health Reimbursements Database
ICPC- 2: The International Classification of Primary Care 2nd Edition
