# Supplementary material for: Utilization of antenatal care among immigrant women in Norway: a nationwide register-based cohort study
Source: BMC Pregnancy Childbirth. 2025 Apr 10;25:417. doi: 10.1186/s12884-025-07519-x (PMC11987317; doi:10.1186/s12884-025-07519-x)
Supplement: Supplementary file 1 — Additional file 1. [file 12884_2025_7519_MOESM1_ESM.docx]

# Additional file 1: Supplemental material

## Tables

**Table A1. Claims registered with other pregnancy-related ICPC-2 codes, additionally included in the sensitivity analyses**

| **ICPC-2 code** | **Explanation** |
| --- | --- |
| W01 | Questions regarding pregnancy |
| W02 | Concerns regarding pregnancy |
| W03 | Bleeding during pregnancy |
| W05 | Nausea during pregnancy |
| W27 | Concerns regarding complications during pregnancy/birth |
| W28 | Reduced functional ability due to pregnancy complications |
| W29 | Symptoms/disorders related to pregnancy/birth |
| W71 | Infection that complicates pregnancy |
| W72 | Malignant tumour associated with pregnancy |
| W73 | Benign/unspecified tumour associated with pregnancy |
| W75 | Injury that complicates pregnancy |
| W76 | Congenital defect that complicates pregnancy |
| W78 | Pregnancy confirmed |
| W81 | Preeclampsia |
| W84 | High-risk pregnancy |
| W85 | Gestational diabetes |

**Table A2: Number of pregnancies, timing of the first antenatal consultation, by country/region of birth**

|  | **Pregnancies, n (col%)** | **First consultation after 13w+6d GA (%)** | **First consultation after week 19w+6d GA (%)** | **Median day of gestation,  first consultation** |
| --- | --- | --- | --- | --- |
| Afghanistan | 1957 (0.6) | 21.9 | 6.1 | 80 |
| Bosnia and Herzegovina | 1326 (0.4) | 15.8 | 5.4 | 74 |
| China | 1273 (0.4) | 17.6 | 5.7 | 76 |
| Eritrea | 2474 (0.7) | 38.1 | 13.6 | 90 |
| Ethiopia | 1302 (0.4) | 28.0 | 10.1 | 82 |
| Europe, Central | 3121 (0.9) | 18.1 | 5.9 | 74 |
| Europe, Eastern | 2638 (0.8) | 20.8 | 6.8 | 78 |
| Europe, Western | 5417 (1.6) | 18.6 | 6.4 | 75 |
| Germany | 1870 (0.5) | 19.7 | 6.6 | 76 |
| High income countries | 1962 (0.6) | 16.1 | 6.2 | 74 |
| India | 1279 (0.4) | 13.6 | 3.8 | 72 |
| Iran | 1504 (0.4) | 17.0 | 5.3 | 75 |
| Iraq | 3510 (1.0) | 21.4 | 7.7 | 76 |
| Kosovo | 1561 (0.4) | 19.0 | 5.8 | 75 |
| Latin America & Caribbean | 2123 (0.6) | 18.7 | 6.5 | 75 |
| Lithuania | 3991 (1.1) | 25.7 | 8.4 | 81 |
| North Africa/Middle East | 1937 (0.6) | 21.4 | 8.0 | 75 |
| Norway | 268 876 (77.1) | 17.0 | 5.7 | 76 |
| Pakistan | 3016 (0.9) | 17.5 | 4.9 | 76 |
| Philippines | 3561 (1.0) | 22.5 | 7.5 | 78 |
| Poland | 7990 (2.3) | 21.5 | 6.8 | 77 |
| Romania | 1560 (0.4) | 24.2 | 7.6 | 77 |
| Russia | 2449 (0.7) | 22.0 | 7.9 | 77 |
| Somalia | 5412 (1.6) | 35.0 | 12.3 | 85 |
| South-East/East Asia & Oceania | 1841 (0.5) | 18.6 | 5.3 | 77 |
| South & Central Asia | 781 (0.2) | 17.9 | 5.9 | 76 |
| Sub-Saharan Africa | 3174 (0.9) | 28.2 | 8.3 | 82 |
| Sweden | 3971 (1.1) | 15.9 | 6.0 | 74 |
| Syria | 1337 (0.4) | 25.6 | 9.6 | 79 |
| Thailand | 2274 (0.7) | 25.3 | 8.3 | 79 |
| Turkey | 1347 (0.4) | 19.6 | 5.1 | 77 |
| Vietnam | 1713 (0.5) | 17.2 | 4.3 | 75 |

Abbreviations: GA = Gestational age

**Table A3. Odds ratio and 95% confidence intervals of risk of not having any antenatal consultations in the 1. trimester.**

|  | **Unadjusted model** | **Adjusted model** |
| --- | --- | --- |
| Intercept | 0.20 (0.20-0.21) | 0.17 (0.17-0.18) |
| **Country/region of origin** |  |  |
| Afghanistan | **1.37 (1.23-1.53)** | 0.87 (0.77-0.99) |
| Bosnia and Herzegovina | 0.92 (0.79-1.07) | 0.87 (0.75-1.01) |
| China | 1.04 (0.90-1.21) | 0.94 (0.80-1.10) |
| Eritrea | **3.01 (2.76-3.28)** | **1.70 (1.53-1.90)** |
| Ethiopia | **1.90 (1.67-2.16)** | 1.24 (1.07-1.43) |
| Europe, Central | 1.08 (0.98-1.19) | 0.89 (0.79-1.00) |
| Europe, Eastern | **1.29 (1.17-1.42)** | 1.07 (0.95-1.21) |
| Europe, Western | 1.12 (1.04-1.20) | 0.90 (0.82-0.98) |
| Germany | 1.20 (1.07-1.36) | 1.03 (0.90-1.18) |
| High income countries | 0.94 (0.83-1.06) | 0.80 (0.70-0.92) |
| India | 0.77 (0.65-0.91) | 0.69 (0.58-0.82) |
| Iran | 1.00 (0.88-1.15) | 0.85 (0.73-0.99) |
| Iraq | **1.33 (1.22-1.45)** | 0.90 (0.81-1.00) |
| Kosovo | 1.15 (1.01-1.30) | 0.92 (0.80-1.05) |
| Latin America & Caribbean | 1.12 (1.00-1.25) | 0.91 (0.80-1.03) |
| Lithuania | **1.69 (1.57-1.82)** | 1.38 (1.25-1.52) |
| North Africa/Middle East | **1.33 (1.19-1.49)** | 0.90 (0.79-1.02) |
| Norway | Ref. | Ref. |
| Pakistan | 1.04 (0.94-1.14) | 0.75 (0.67-0.84) |
| Philippines | **1.42 (1.31-1.54)** | 1.20 (1.09-1.33) |
| Poland | **1.34 (1.27-1.42)** | 1.11 (1.02-1.21) |
| Romania | **1.56 (1.38-1.78)** | 1.23 (1.06-1.41) |
| Russia | **1.38 (1.25-1.53)** | 1.09 (0.97-1.23) |
| Somalia | **2.63 (2.48-2.79)** | 1.28 (1.18-1.40) |
| South-East/East Asia & Oceania | 1.12 (0.99-1.27) | 0.91 (0.80-1.05) |
| South & Central Asia | 1.07 (0.89-1.28) | 0.87 (0.71-1.05) |
| Sub-Saharan Africa | **1.92 (1.77-2.08)** | 1.20 (1.08-1.33) |
| Sweden | 0.93 (0.85-1.01) | 0.81 (0.73-0.91) |
| Syria | **1.68 (1.49-1.90)** | 0.82 (0.71-0.94) |
| Thailand | **1.66 (1.51-1.83)** | 1.27 (1.13-1.42) |
| Turkey | 1.19 (1.04-1.37) | 0.90 (0.78-1.04) |
| Vietnam | 1.01 (0.89-1.15) | 0.84 (0.73-0.96) |
| **Birth year** |  |  |
| 2012 |  | Ref. |
| 2013 |  | 1.02 (0.99-1.05) |
| 2014 |  | 1.03 (0.99-1.06) |
| 2015 |  | 1.06 (1.02-1.09) |
| 2016 |  | 1.10 (1.06-1.13) |
| 2017 |  | 1.15 (1.11-1.19) |
| 2018 |  | 1.10 (1.07-1.14) |
| **Parity** |  |  |
| 0 children |  | 0.83 (0.81-0.85) |
| 1 child |  | Ref. |
| 2 children |  | 1.33 (1.30-1.37) |
| 3 children |  | 1.77 (1.69-1.84) |
| ≥4 children |  | 2.31 (2.18-2.45) |
| **Age** |  |  |
| 16-20 years |  | 1.55 (1.46-1.64) |
| 21-25 years |  | 1.09 (1.05-1.12) |
| 26-30 years |  | 0.98 (0.95-1.00) |
| 31-35 years |  | Ref. |
| ≥36 years |  | 1.16 (1.13-1.20) |
| **Civil status** |  |  |
| Married/registered partner |  | Ref. |
| Cohabiting |  | 1.07 (1.05-1.09) |
| No partner |  | 1.36 (1.31-1.42) |
| Other/unknown |  | 1.19 (1.10-1.29) |
| **Educational attainment** |  |  |
| Primary school |  | Ref. |
| Upper secondary school |  | 0.92 (0.89-0.94) |
| Bachelor’s degree or equivalent |  | 0.86 (0.83-0.88) |
| Masters’ degree or equivalent |  | 0.85 (0.82-0.88) |
| Missing |  | 1.02 (0.98-1.07) |
| **Household income** |  |  |
| Lowest quartile |  | Ref. |
| Mid-lowest quartile |  | 0.88 (0.86-0.91) |
| Mid-highest quartile |  | 0.87 (0.84-0.89) |
| Highest quartile |  | 0.92 (0.89-0.95) |
| **Partner’s origin** |  |  |
| Norwegian-born |  | Ref. |
| Foreign-born |  | 1.01 (0.98-1.04) |
| Missing |  | 1.52 (1.44-1.60) |
| **Period of residency** |  |  |
| ≤2 years |  | 1.42 (1.32-1.53) |
| >2 - ≤5 years |  | 1.18 (1.10-1.27) |
| >5 - ≤10 years |  | 1.08 (1.01-1.16) |
| >10 - ≤15 years |  | 1.00 (0.92-1.09) |
| >15 years or Norwegian origin |  | Ref. |

**Table A4. Odds ratio and 95% confidence intervals of risk of having fewer antenatal consultations than recommended in the 2. trimester.**

|  | **Unadjusted model** | **Adjusted model** |
| --- | --- | --- |
| Intercept | 0.07 (0.07-0.07) | 0.08 (0.08-0.09) |
| **Country/region of origin** |  |  |
| Afghanistan | 0.85 (0.70-1.04) | 0.69 (0.55-0.87) |
| Bosnia and Herzegovina | 0.77 (0.60-0.98) | 0.77 (0.60-1.00) |
| China | 0.80 (0.62-1.03) | 0.77 (0.58-1.01) |
| Eritrea | 1.07 (0.91-1.26) | 0.89 (0.73-1.08) |
| Ethiopia | 0.88 (0.70-1.11) | 0.72 (0.56-0.93) |
| Europe, Central | 1.00 (0.86-1.15) | 0.95 (0.80-1.13) |
| Europe, Eastern | 1.19 (1.03-1.37) | 1.11 (0.93-1.33) |
| Europe, Western | 1.15 (1.03-1.28) | 1.04 (0.90-1.20) |
| Germany | **1.31 (1.10-1.55)** | 1.21 (1.00-1.48) |
| High income countries | **1.31 (1.11-1.54**) | **1.25 (1.04-1.49)** |
| India | 0.55 (0.41-0.74) | 0.55 (0.40-0.75) |
| Iran | 0.70 (0.55-0.90) | 0.67 (0.52-0.88) |
| Iraq | 1.05 (0.91-1.19) | 0.85 (0.72-1.01) |
| Kosovo | 0.89 (0.72-1.10) | 0.82 (0.65-1.03) |
| Latin America & Caribbean | 0.92 (0.76-1.10) | 0.85 (0.69-1.04) |
| Lithuania | 0.96 (0.84-1.09) | 0.89 (0.75-1.06) |
| North Africa/Middle East | 1.22 (1.04-1.45) | 1.02 (0.84-1.23) |
| Norway | Ref. | Ref. |
| Pakistan | 0.88 (0.76-1.03) | 0.76 (0.64-0.91) |
| Philippines | 1.15 (1.01-1.31) | 1.13 (0.96-1.33) |
| Poland | 1.06 (0.97-1.16) | 1.00 (0.87-1.15) |
| Romania | 0.91 (0.74-1.12) | 0.84 (0.67-1.07) |
| Russia | 1.28 (1.11-1.49) | 1.13 (0.94-1.35) |
| Somalia | **1.40 (1.27-1.54)** | 0.93 (0.80-1.07) |
| South-East/East Asia & Oceania | 0.78 (0.63-0.97) | 0.73 (0.58-0.92) |
| South & Central Asia | 0.75 (0.54-1.03) | 0.70 (0.50-0.99) |
| Sub-Saharan Africa | 0.95 (0.82-1.10) | 0.76 (0.63-0.90) |
| Sweden | 0.99 (0.87-1.13) | 0.96 (0.82-1.14) |
| Syria | 0.94 (0.75-1.17) | 0.79 (0.61-1.02) |
| Thailand | 1.18 (1.01-1.38) | 1.08 (0.90-1.30) |
| Turkey | 0.79 (0.62-1.01) | 0.70 (0.54-0.91) |
| Vietnam | 0.73 (0.59-0.91) | 0.67 (0.53-0.85) |
| **Birth year** |  |  |
| 2012 |  | Ref. |
| 2013 |  | 0.88 (0.84-0.92) |
| 2014 |  | 0.86 (0.82-0.90) |
| 2015 |  | 0.73 (0.70-0.77) |
| 2016 |  | 0.72 (0.68-0.75) |
| 2017 |  | 0.68 (0.64-0.71) |
| 2018 |  | 0.55 (0.52-0.58) |
| **Parity** |  |  |
| 0 children |  | 0.85 (0.82-0.88) |
| 1 child |  | Ref. |
| 2 children |  | 1.17 (1.13-1.22) |
| 3 children |  | 1.31 (1.23-1.40) |
| ≥4 children |  | 1.54 (1.40-1.69) |
| **Age** |  |  |
| 16-20 years |  | 1.29 (1.17-1.42) |
| 21-25 years |  | 1.09 (1.04-1.15) |
| 26-30 years |  | 0.99 (0.95-1.02) |
| 31-35 years |  | Ref. |
| ≥36 years |  | 0.99 (0.95-1.03) |
| **Civil status** |  |  |
| Married/registered partner |  | Ref. |
| Cohabiting |  | 1.04 (1.01-1.08) |
| No partner |  | 1.02 (0.96-1.10) |
| Other/unknown |  | 1.02 (0.89-1.16) |
| **Educational attainment** |  |  |
| Primary school |  | Ref. |
| Upper secondary school |  | 0.95 (0.91-1.00) |
| Bachelor’s degree or equivalent |  | 0.99 (0.95-1.04) |
| Masters’ degree or equivalent |  | 1.17 (1.11-1.23) |
| Missing |  | 1.13 (1.05-1.21) |
| **Household income** |  |  |
| Lowest quartile |  | Ref. |
| Mid-lowest quartile |  | 0.91 (0.87-0.94) |
| Mid-highest quartile |  | 0.86 (0.83-0.90) |
| Highest quartile |  | 0.89 (0.85-0.93) |
| **Partner’s origin** |  |  |
| Norwegian-born |  | Ref. |
| Foreign-born |  | 0.97 (0.93-1.02) |
| Missing |  | 1.74 (1.61-1.89) |
| **Period of residency** |  |  |
| ≤2 years |  | 1.13 (1.00-1.26) |
| >2 - ≤5 years |  | 1.07 (0.96-1.20) |
| >5 - ≤10 years |  | 0.96 (0.86-1.08) |
| >10 - ≤15 years |  | 1.04 (0.91-1.18) |
| >15 years or Norwegian origin |  | Ref. |

**Table A5. Odds ratio and 95% confidence intervals of risk of having fewer antenatal consultations than recommended in the 3. trimester.**

|  | **Unadjusted model** | **Adjusted model** |
| --- | --- | --- |
| Intercept | 0.29 (0.29-0.29) | 0.46 (0.44-0.48) |
| **Country/region of origin** |  |  |
| Afghanistan | 0.95 (0.85-1.07) | 0.79 (0.69-0.90) |
| Bosnia and Herzegovina | 0.85 (0.74-0.98) | 0.85 (0.74-0.99) |
| China | 0.71 (0.61-0.82) | 0.73 (0.62-0.86) |
| Eritrea | 1.04 (0.94-1.15) | 0.90 (0.80-1.01) |
| Ethiopia | 0.80 (0.69-0.93) | 0.70 (0.60-0.82) |
| Europe, Central | 1.05 (0.96-1.14) | 1.06 (0.96-1.18) |
| Europe, Eastern | 1.07 (0.97-1.17) | 1.09 (0.97-1.22) |
| Europe, Western | 1.09 (1.02-1.17) | 1.07 (0.98-1.17) |
| Germany | **1.29 (1.16-1.44)** | **1.29 (1.14-1.45)** |
| High income countries | 1.21 (1.09-1.35) | 1.20 (1.07-1.35) |
| India | 0.74 (0.63-0.86) | 0.77 (0.65-0.91) |
| Iran | 0.90 (0.79-1.02) | 0.88 (0.77-1.01) |
| Iraq | 1.17 (1.08-1.27) | 0.96 (0.87-1.06) |
| Kosovo | 1.28 (1.14-1.44) | 1.17 (1.03-1.33) |
| Latin America & Caribbean | 1.03 (0.93-1.15) | 1.00 (0.89-1.13) |
| Lithuania | 0.97 (0.90-1.05) | 1.01 (0.91-1.12) |
| North Africa/Middle East | 1.25 (1.13-1.40) | 1.06 (0.94-1.20) |
| Norway | Ref. | Ref. |
| Pakistan | 1.11 (1.02-1.21) | 0.95 (0.86-1.05) |
| Philippines | 0.93 (0.86-1.01) | 0.96 (0.87-1.06) |
| Poland | 1.17 (1.11-1.24) | 1.20 (1.10-1.30) |
| Romania | 0.98 (0.87-1.11) | 0.98 (0.85-1.12) |
| Russia | **1.29 (1.18-1.42)** | 1.21 (1.09-1.35) |
| Somalia | **1.39 (1.30-1.48)** | 0.99 (0.91-1.09) |
| South-East/East Asia & Oceania | 0.89 (0.79-1.00) | 0.83 (0.73-0.94) |
| South & Central Asia | 1.03 (0.87-1.22) | 1.05 (0.87-1.26) |
| Sub-Saharan Africa | 1.13 (1.04-1.23) | 0.95 (0.86-1.06) |
| Sweden | 0.96 (0.89-1.04) | 1.02 (0.92-1.12) |
| Syria | 1.21 (1.07-1.37) | 1.02 (0.88-1.17) |
| Thailand | 1.14 (1.03-1.26) | 1.07 (0.95-1.20) |
| Turkey | 1.06 (0.93-1.20) | 0.90 (0.79-1.04) |
| Vietnam | 0.79 (0.70-0.90) | 0.72 (0.63-0.83) |
| **Birth year** |  |  |
| 2012 |  | Ref. |
| 2013 |  | 0.88 (0.85-0.90) |
| 2014 |  | 0.86 (0.84-0.89) |
| 2015 |  | 0.78 (0.76-0.81) |
| 2016 |  | 0.77 (0.75-0.79) |
| 2017 |  | 0.77 (0.75-0.79) |
| 2018 |  | 0.73 (0.70-0.75) |
| **Parity** |  |  |
| 0 children |  | 0.73 (0.71-0.75) |
| 1 child |  | Ref. |
| 2 children |  | 1.16 (1.13-1.19) |
| 3 children |  | 1.30 (1.25-1.36) |
| ≥4 children |  | 1.49 (1.41-1.58) |
| **Age** |  |  |
| 16-20 years |  | 1.09 (1.03-1.16) |
| 21-25 years |  | 1.00 (0.97-1.03) |
| 26-30 years |  | 0.96 (0.94-0.98) |
| 31-35 years |  | Ref. |
| ≥36 years |  | 1.09 (1.06-1.12) |
| **Civil status** |  |  |
| Married/registered partner |  | Ref. |
| Cohabiting |  | 0.98 (0.96-1.00) |
| No partner |  | 1.04 (1.00-1.09) |
| Other/unknown |  | 1.07 (0.99-1.16) |
| **Educational attainment** |  |  |
| Primary school |  | Ref. |
| Upper secondary school |  | 0.90 (0.87-0.92) |
| Bachelor’s degree or equivalent |  | 0.87 (0.84-0.89) |
| Masters’ degree or equivalent |  | 0.92 (0.89-0.95) |
| Missing |  | 0.98 (0.94-1.03) |
| **Household income** |  |  |
| Lowest quartile |  | Ref. |
| Mid-lowest quartile |  | 0.91 (0.89-0.93) |
| Mid-highest quartile |  | 0.88 (0.86-0.91) |
| Highest quartile |  | 0.92 (0.90-0.95) |
| **Partner’s origin** |  |  |
| Norwegian-born |  | Ref. |
| Foreign-born |  | 1.01 (0.98-1.03) |
| Missing |  | 1.22 (1.16-1.28) |
| **Period of residency** |  |  |
| ≤2 years |  | 0.95 (0.88-1.01) |
| >2 - ≤5 years |  | 0.97 (0.90-1.03) |
| >5 - ≤10 years |  | 0.94 (0.88-1.00) |
| >10 - ≤15 years |  | 0.97 (0.90-1.04) |
| >15 years or Norwegian origin |  | Ref. |

## Figures


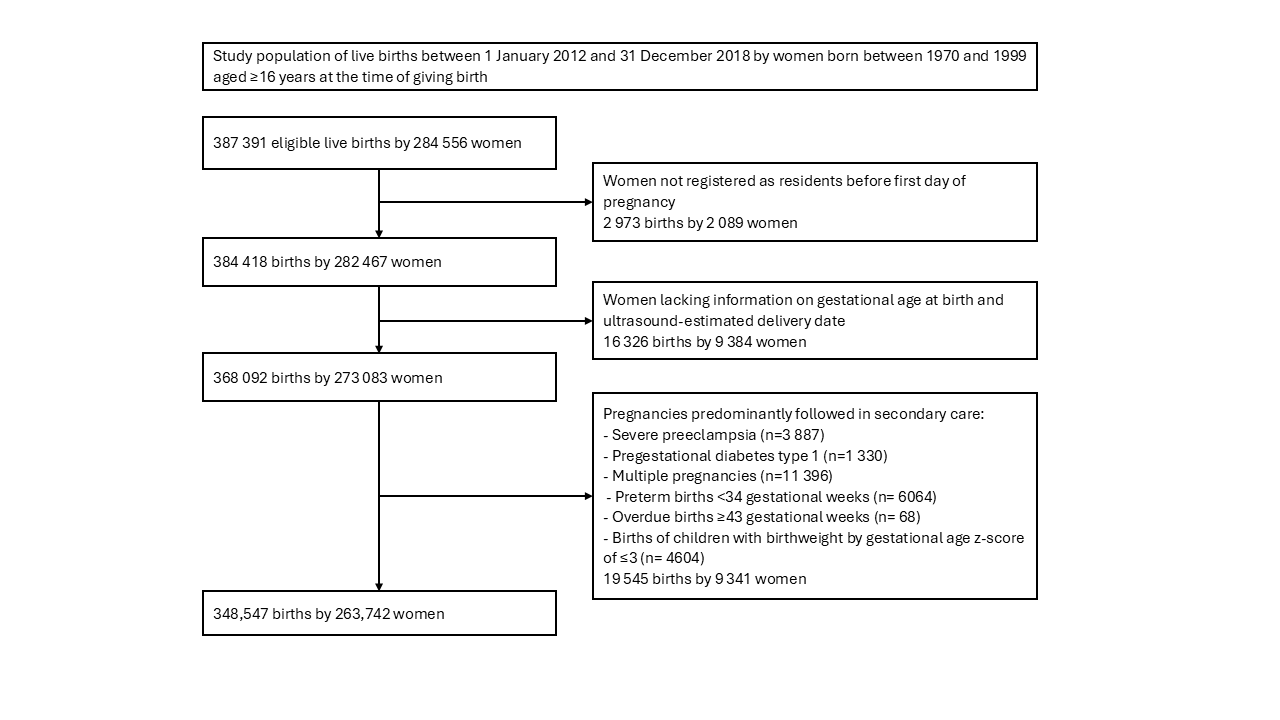


Figure A1. Flow chart of study population.


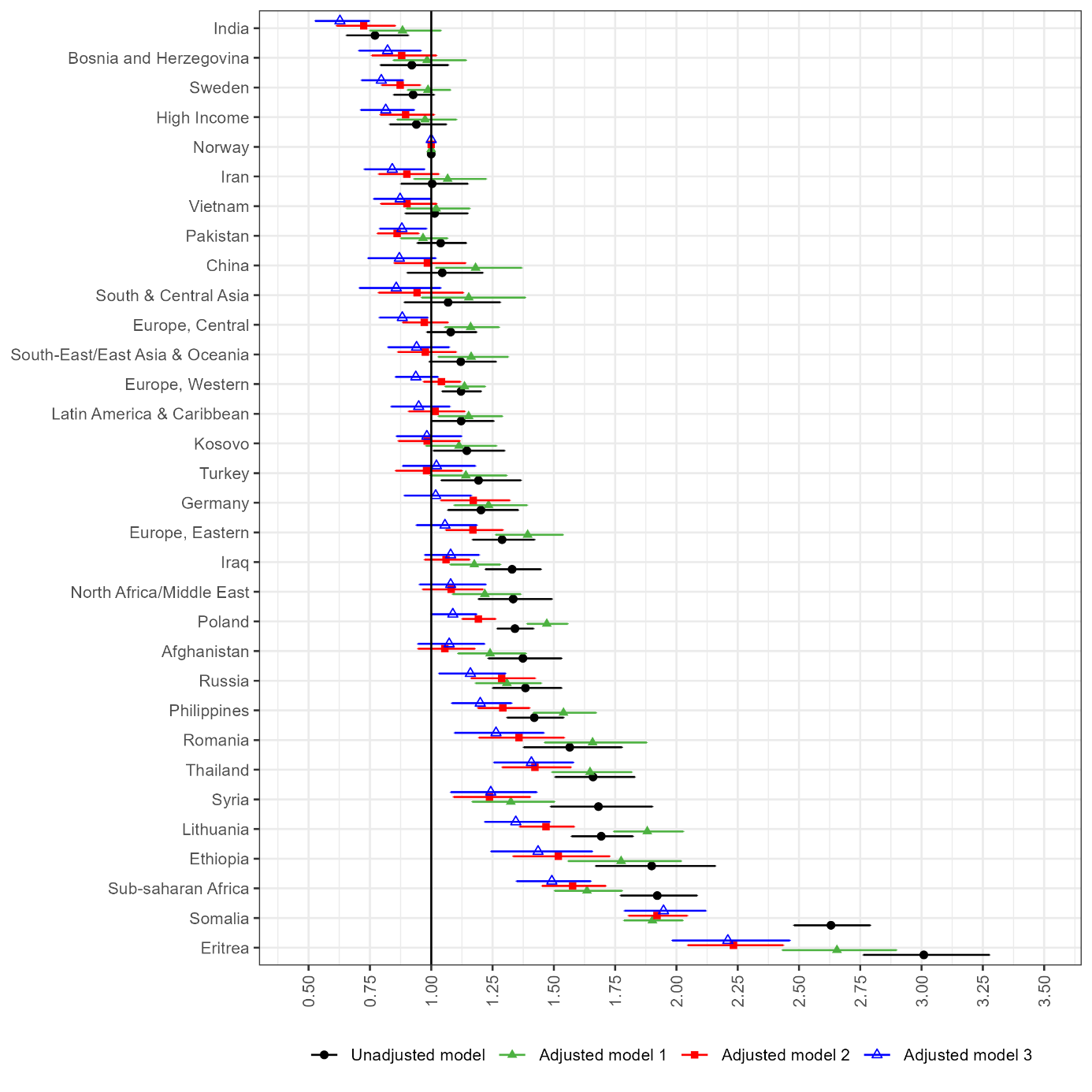


Figure A2. Odds ratios and 95% confidence intervals of not having any antenatal consultations during the first trimester compared with Norwegian women, by country/region of birth. Adjusted model 1 includes child birth year, parity, age, civil status, Adjusted model 2 includes maternal educational attainment and household income, and Adjusted model 3 includes partner’s origin and period of residency.


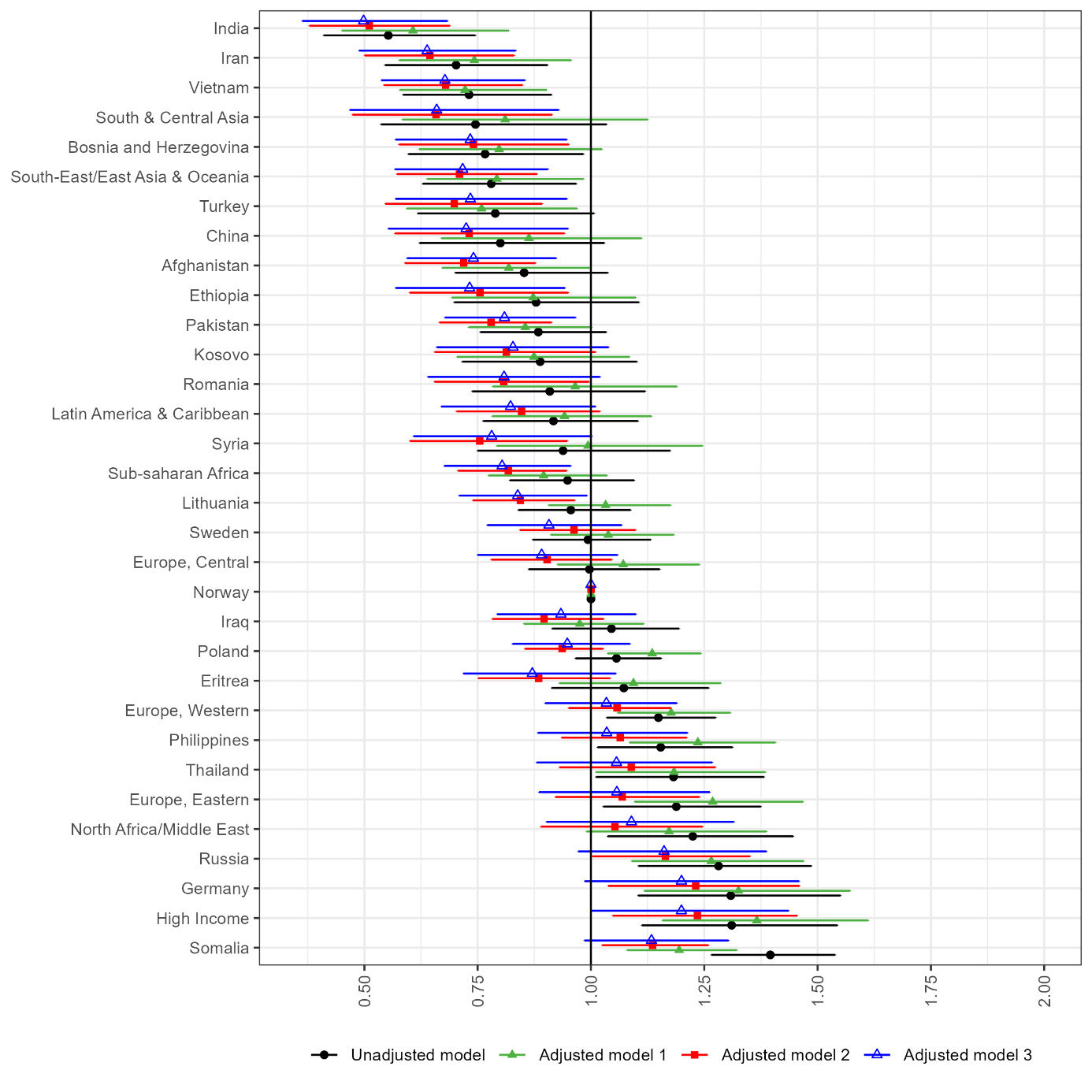


Figure A3. Odds ratios and 95% confidence intervals of having fewer consultations than recommended during the second trimester compared with Norwegian women, by country/region of birth. Adjusted model 1 includes child birth year, parity, age, civil status, Adjusted model 2 includes maternal educational attainment and household income, and Adjusted model 3 includes partner’s origin and period of residency.


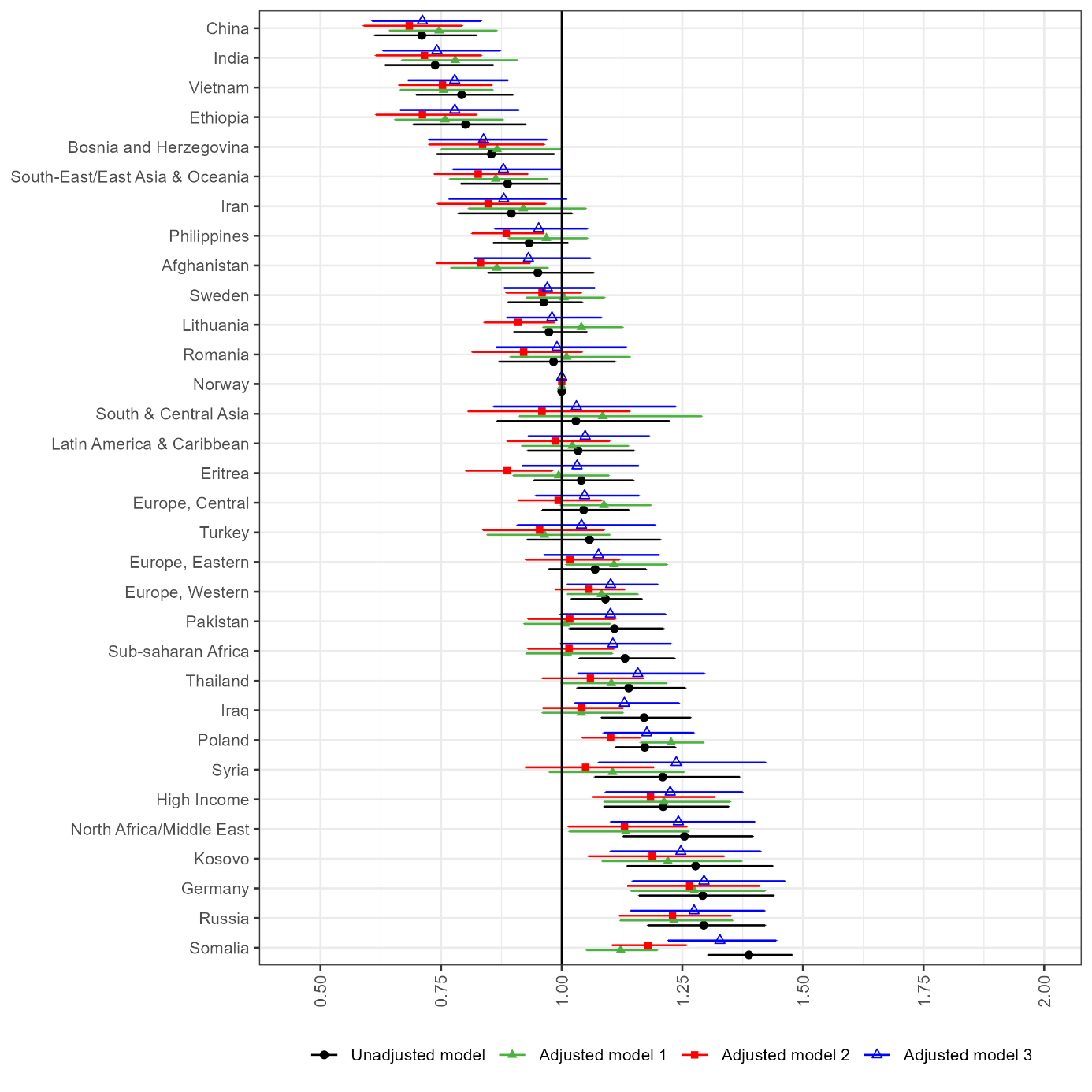


Figure A4. Odds ratios and 95% confidence intervals of having fewer consultations than recommended during the third trimester compared with Norwegian women, by country/region of birth. Adjusted model 1 includes child birth year, parity, age, civil status, Adjusted model 2 includes maternal educational attainment and household income, and Adjusted model 3 includes partner’s origin and period of residency.


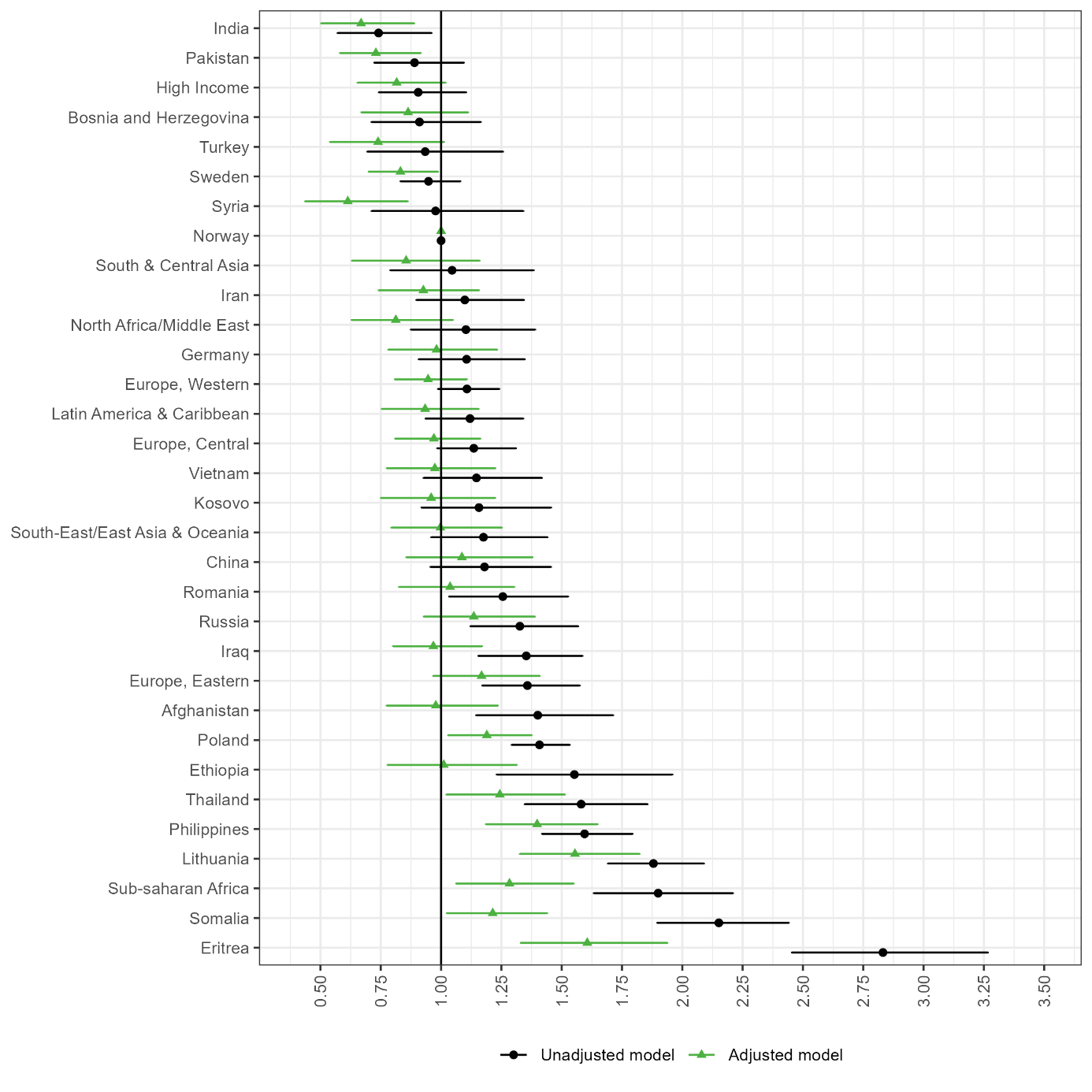


Figure A5. Odds ratios and 95% confidence intervals of not having any antenatal consultations during the first trimester among nulliparous women compared with Norwegian women, by country/region of birth. The adjusted model includes child birth year, parity, age, civil status, maternal educational attainment, household income, partner’s origin and period of residency.


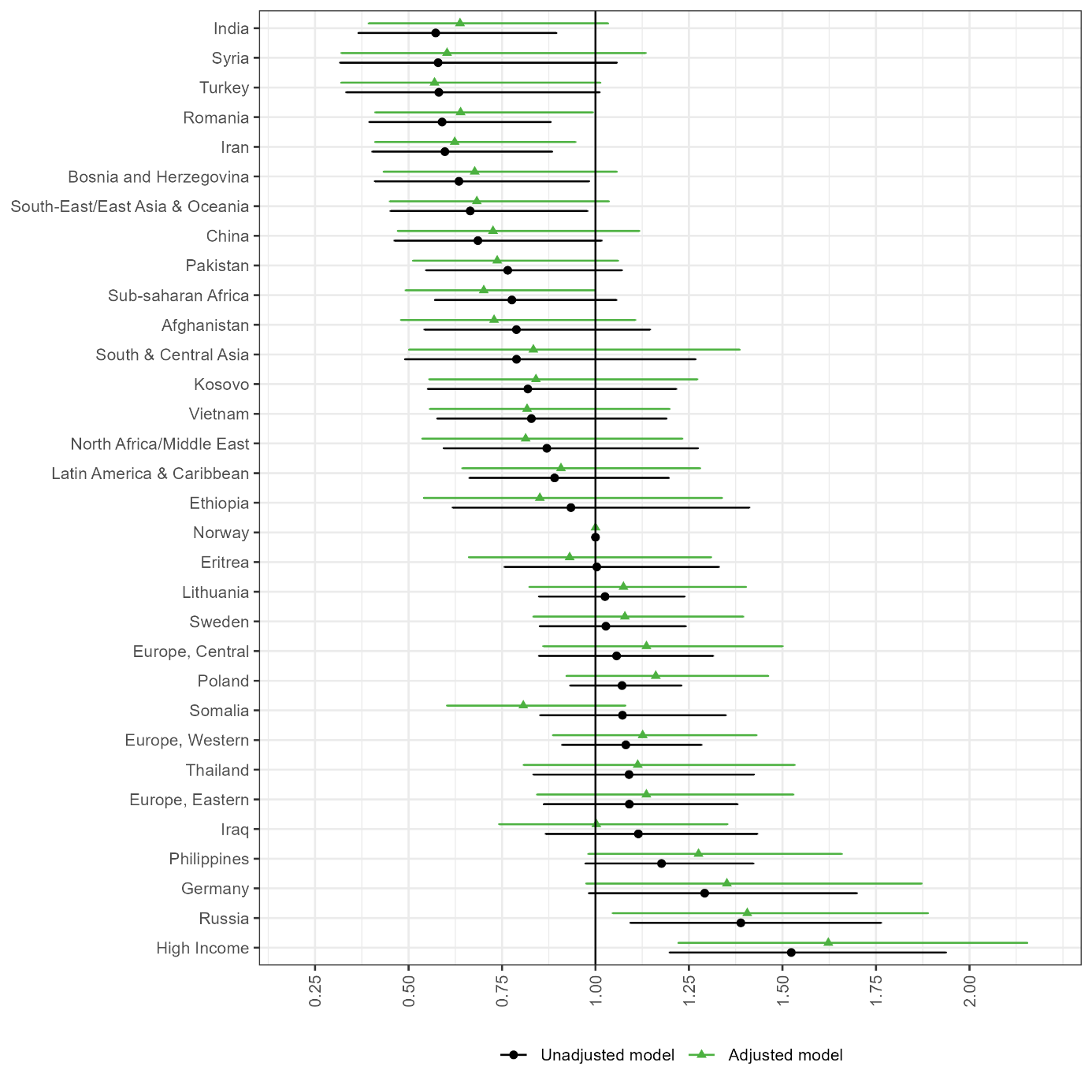


Figure A6. Odds ratios and 95% confidence intervals of having fewer consultations than recommended during the second trimester among nulliparous women compared to Norwegian women, by country/region of birth. The adjusted model includes child birth year, parity, age, civil status, maternal educational attainment, household income, partner’s origin and period of residency.


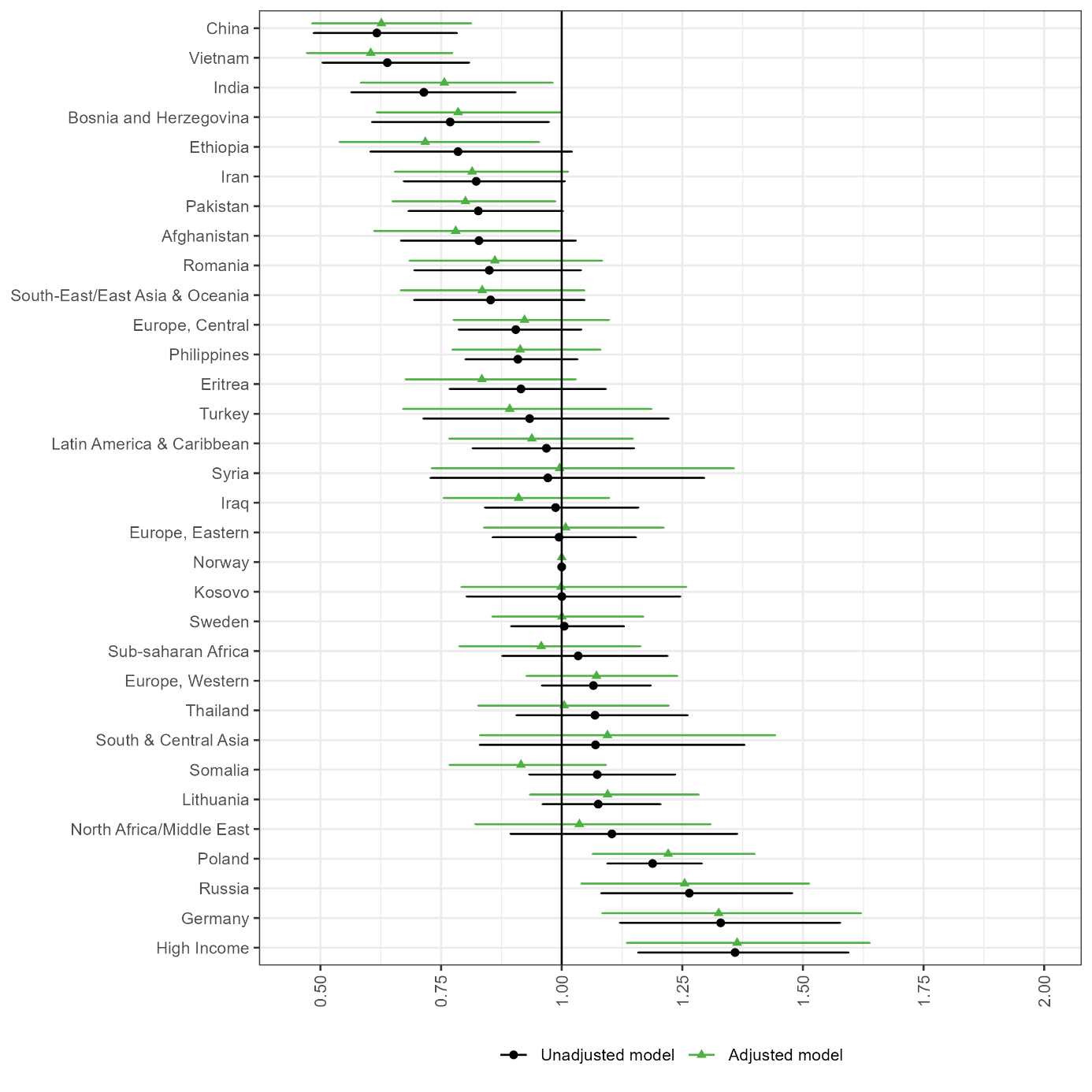


Figure A7. Odds ratios and 95% confidence intervals of having fewer consultations than recommended during the third trimester among nulliparous women compared to Norwegian women by country/region of birth. The adjusted model includes child birth year, parity, age, civil status, maternal educational attainment, household income, partner’s origin and period of residency.


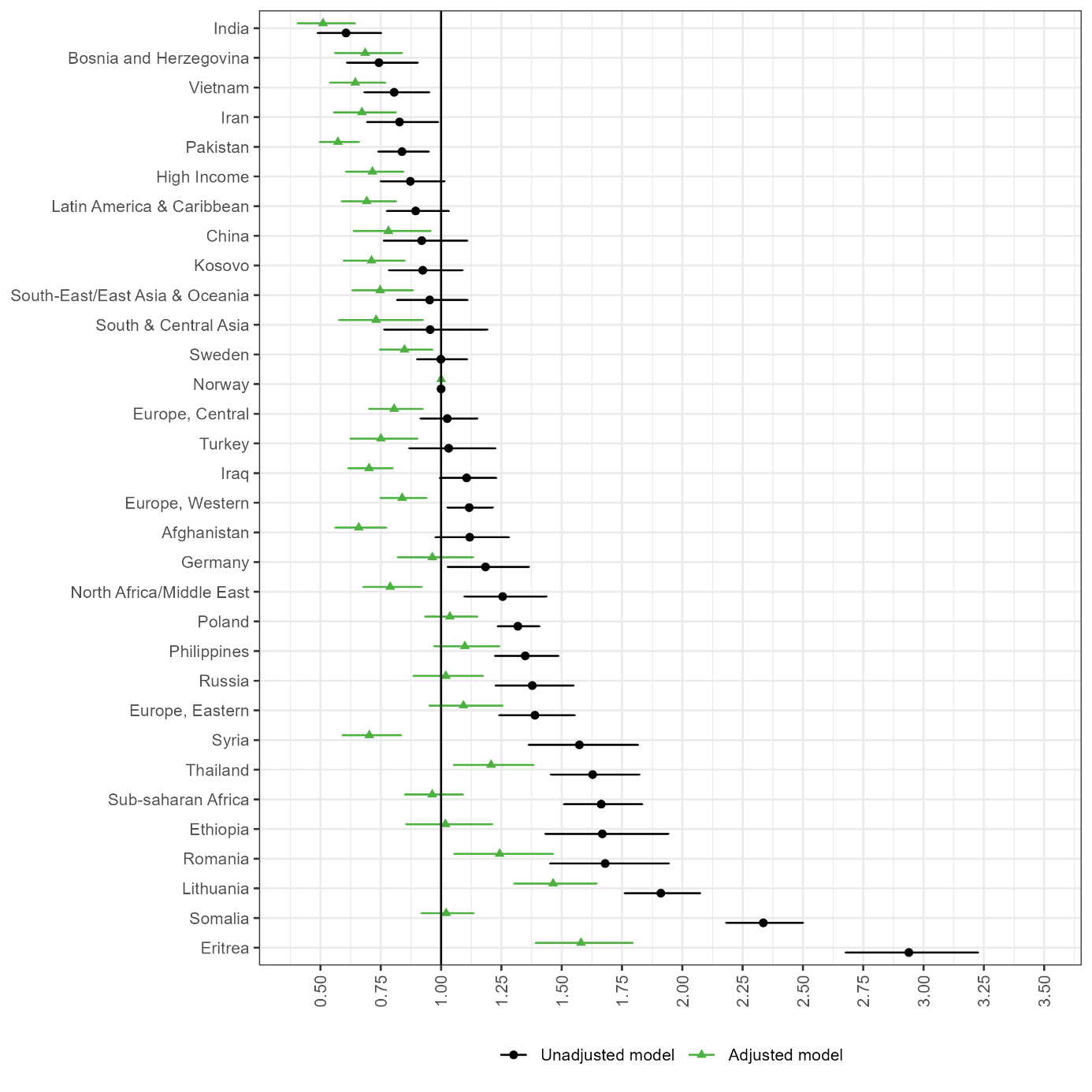


Figure A8. Odds ratios and 95% confidence intervals of not having any antenatal consultations during the first trimester compared to Norwegian women, by country/region of birth, when including additional pregnancy-related diagnosis codes. The adjusted model includes child birth year, parity, age, civil status, maternal educational attainment, household income, partner’s origin and period of residency.


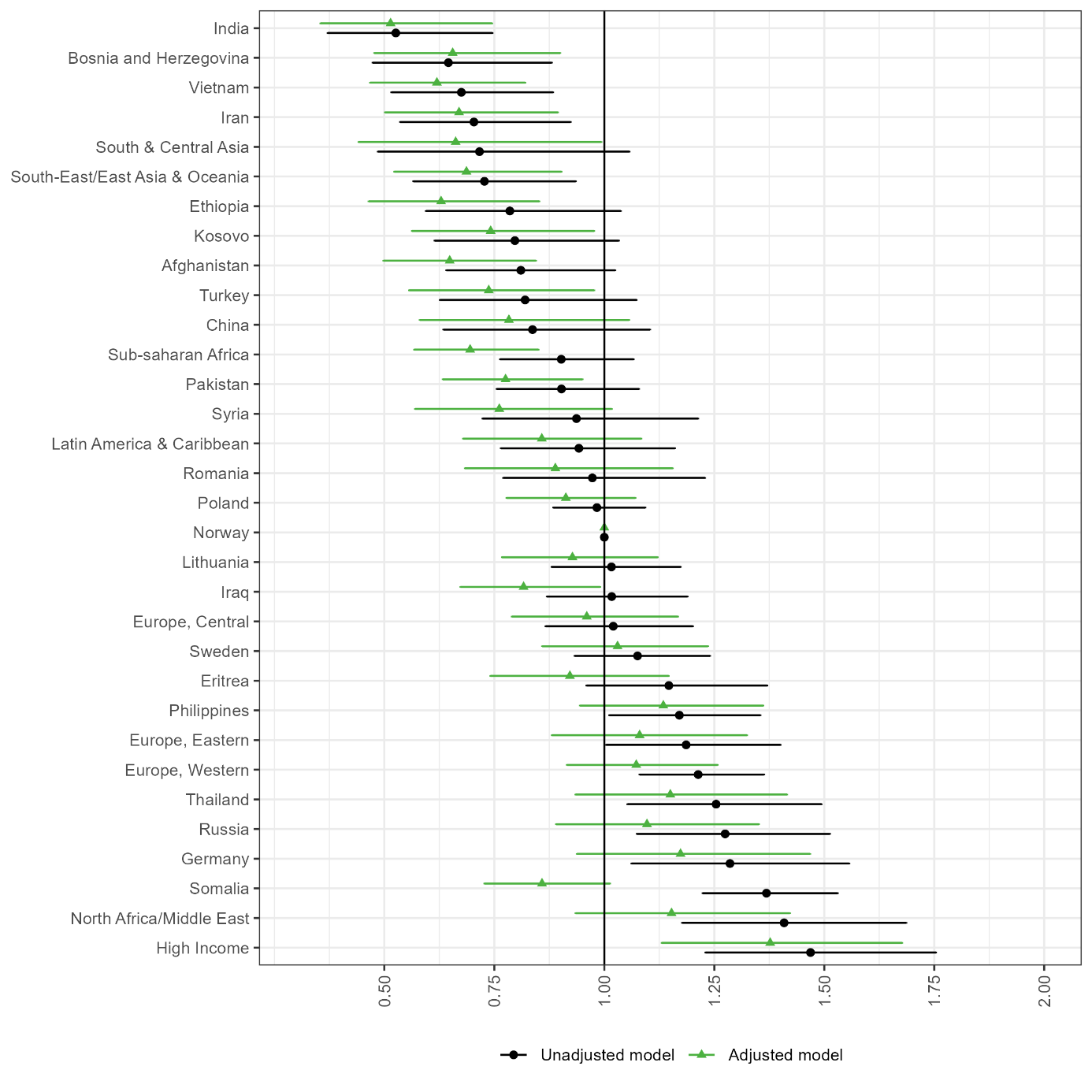


Figure A9. Odds ratios and 95% confidence intervals of having fewer consultations than recommended during the second trimester compared to Norwegian women, by country/region of birth, when including additional pregnancy-related diagnosis codes. The adjusted model includes child birth year, parity, age, civil status, maternal educational attainment, household income, partner’s origin and period of residency.


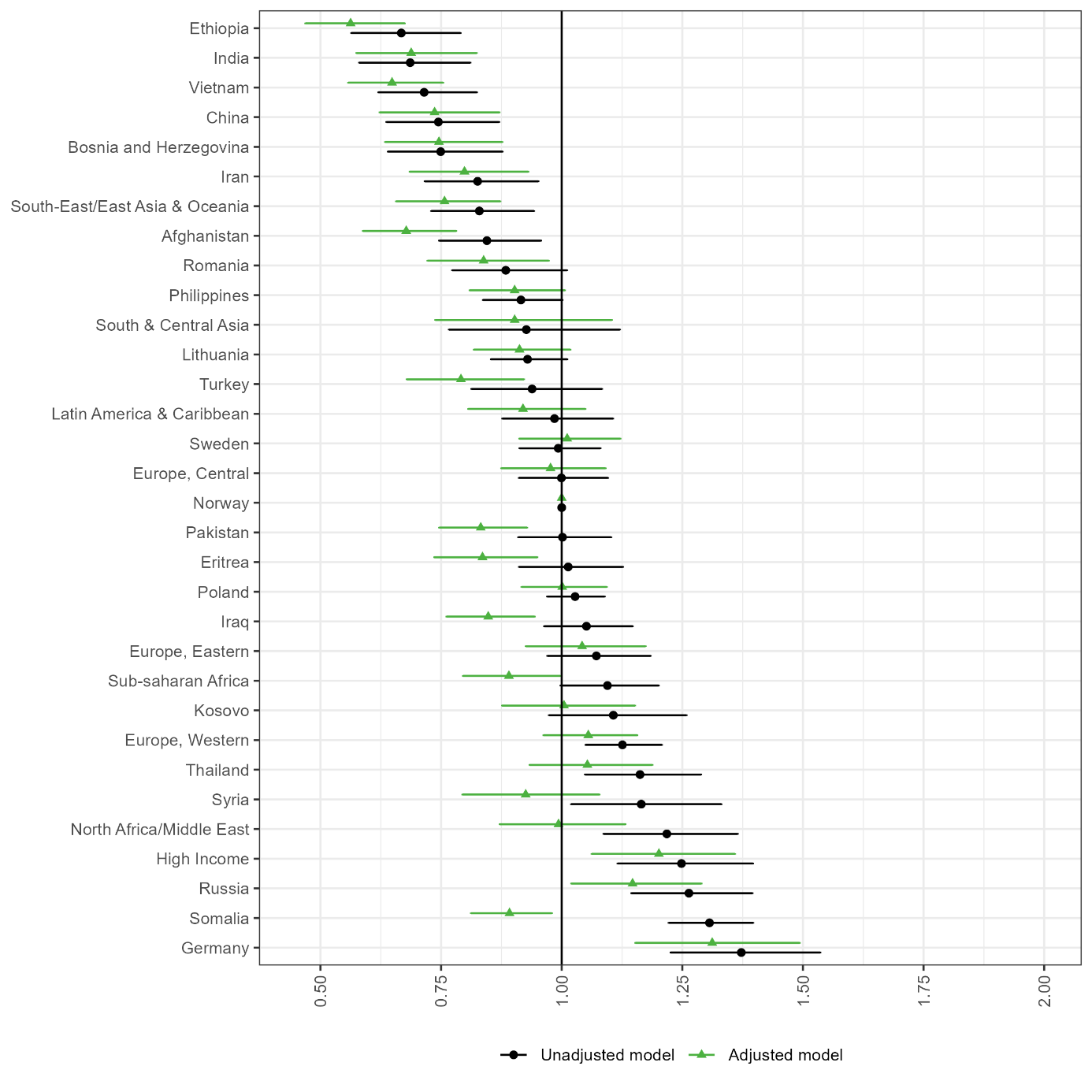


Figure A10. Odds ratios and 95% confidence intervals of having fewer consultations than recommended during the third trimester compared to Norwegian women, by country/region of birth, when including additional pregnancy-related diagnosis codes. The adjusted model includes child birth year, parity, age, civil status, maternal educational attainment, household income, partner’s origin and period of residency.
